# Investigating New Particle Formation and Growth Over an Urban Location in the Eastern Mediterranean

**DOI:** 10.1029/2024JD041802

**Published:** 2024-11-30

**Authors:** T. C. Ajith, Eli Windwer, Chunlin Li, Zheng Fang, Sobhan Kumar Kompalli, Farhan R. Nursanto, Temitope E. Olayemi, Julius I. Ese, Steven A. L. Sharpe, Matthew Fraund, Ryan C. Moffet, Alexander Laskin, Juliane L. Fry, Yinon Rudich

**Affiliations:** ^1^ Department of Earth and Planetary Sciences Weizmann Institute of Science Rehovot Israel; ^2^ College of Environmental Science and Engineering Tongji University Shanghai China; ^3^ Space Physics Laboratory Vikram Sarabhai Space Centre Thiruvananthapuram India; ^4^ Meteorology and Air Quality (MAQ) Environmental Sciences Group Wageningen University and Research (WUR) Wageningen The Netherlands; ^5^ Department of Chemistry Purdue University West Lafayette IN USA; ^6^ Fraund Consulting Pleasant Hill CA USA; ^7^ Sonoma Technology, Inc. Petaluma CA USA; ^8^ Department of Earth Atmospheric and Planetary Sciences Purdue University West Lafayette IN USA

**Keywords:** new particle formation, hybrid PMF analysis, nighttime NPF

## Abstract

This study investigates the new particle formation (NPF) events at an urban location in the Eastern Mediterranean. Particle size distribution, particulate chemical composition, and gaseous pollutants were monitored in Rehovot, Israel (31°53″N 34°48″E) during two campaigns: from April 29 to 3 May 2021 (Campaign 1) and from May 3 to 11 May 2023 (Campaign 2), coinciding with an intensive bonfire burning festival. The organic aerosols (OA) source apportionment identified two major factors—Hydrocarbon‐like OA and Biomass‐burning OA—as well as two secondary factors—MO‐OOA (more oxidized‐oxygenated OA) and LO‐OOA (low oxidized oxygenated OA). NPF events were frequently observed during the day (mostly well‐defined nucleation events) and at night (burst of ultrafine mode particles without any discernible growth). A condensation sink value of (9.4 ± 4.0) × 10^−3^ s^−1^ during Campaign 1 and (14.2 ± 6.0) × 10^−3^ s^−1^ during Campaign 2 was obtained. The daytime events were associated with enhanced sulfuric acid proxy concentrations of (2–12) × 10^6^ molecules cm^−3^, suggesting the role of gas‐phase photochemistry in promoting NPF. A novel approach of hybrid positive matrix factorization analysis was used to deconvolve the chemical species responsible for the observed events. The results suggest the involvement of multiple components, including ammonium sulfate and MO‐OOA, in the nucleation; Nitrate, HOA and LO‐OOA participate in the subsequent particle growth for the daytime events. Nighttime events involve only semi‐volatile species (LO‐OOA, HOA and nitrate) along with ammonium sulfate.

## Key Points

- New particle formation events were investigated in the Eastern Mediterranean
- Sulfuric acid and more oxidized‐oxygenated organic aerosols trigger the daytime NPF
- Nighttime events are mostly associated with semi‐volatile species

## 1 Introduction

Atmospheric aerosols are critical in regulating Earth's radiative balance, and climate (Forster et al., 2023), while adversely affecting visibility, air quality (Yao et al., 2021; Q. Zhang et al., 2015), and human health (Bu et al., 2021). Their direct interaction with incoming solar radiation through absorption and scattering significantly impacts Earth's radiation budget (Bellouin et al., 2020; Levy et al., 2013; Stier et al., 2007). Additionally, aerosols are nuclei for cloud condensation and ice formation, influencing cloud properties, including formation, albedo, lifetime, and precipitation (Lohmann & Feichter, 2005; Rosenfeld et al., 2008). Notably, substantial uncertainties persist in understanding and quantifying the effects of these interactions (IPCC, 2022). A thorough evaluation of their influence necessitates acquiring an in‐depth understanding of aerosol characteristics, including concentrations, size distributions, chemical compositions, and the underlying dynamical processes governing their evolution. New particle formation (NPF) from gaseous precursors is a critical process in this regard since it generates a significant number of aerosol particles (Kerminen et al., 2018) and contributes to nearly half of the concentration of cloud condensation nuclei (CCN) (Merikanto et al., 2009). NPF and subsequent growth processes modify the atmospheric environment, influencing aerosol‐cloud interactions, which makes studying NPF in diverse atmospheric conditions critical (Sebastian et al., 2022).

The initial step in NPF is the formation of nanometer‐sized particles through the agglomeration of molecular clusters, which grow into detectable nucleation‐mode particles (<25 nm) (Kulmala et al., 2012, 2014). Subsequently, newly formed particles can grow to Aitken‐mode (25–100 nm) and larger sizes, acting as potential CCN (Kulmala et al., 2014; R. Zhang, 2010). The typical precursors for cluster formation are extremely low‐volatility compounds such as sulfuric acid, highly oxygenated organic molecules, and iodic acids (Bianchi et al., 2016; Kirkby et al., 2016, 2023; Sipilä et al., 2010; Tröstl et al., 2016). While the initial molecular clustering is widely believed to occur everywhere, further growth via homogeneous or heterogeneous processes requires specific atmospheric conditions (Kulmala et al., 2013, 2022; Lee et al., 2019; Tröstl et al., 2016). Pre‐existing particles can inhibit NPF events by acting as a sink for the molecular clusters and the low‐volatility gas phase species (Kompalli et al., 2014, 2018, 2020; Kulmala et al., 2017; Tuovinen et al., 2020). Further, the intensity of solar radiation, relative humidity (RH), and atmospheric mixing conditions affect the abundance and properties of gas‐phase species, molecular clusters, and pre‐existing particles; and hence, NPF.

In recent years, substantial efforts have been made to understand the mechanism of atmospheric NPF events, including field studies (Bianchi et al., 2016; Chu et al., 2019; Kerminen et al., 2018; Kulmala et al., 2013, 2023), laboratory experiments (Kirkby et al., 2023; Riccobono et al., 2012; Tröstl et al., 2016; M. Wang et al., 2020), and theoretical calculations (Kulmala et al., 2017; Riipinen et al., 2011; R. Zhang et al., 2012). NPF is frequently observed in distinct environmental conditions, such as the free‐troposphere (Bianchi et al., 2016), urban (Chu et al., 2019), semi‐urban areas (Zhu et al., 2021), coastal regions (O’Dowd et al., 2002), remote locations (Bousiotis et al., 2021), industrial sites (Junkermann et al., 2011), and forested areas (Held et al., 2004; Kanawade et al., 2011). It has been reported that many chemical compounds and precursors influence NPF; for example, sulfuric acid (Kulmala & Kerminen, 2008; Kulmala, Vehkamäki, et al., 2004; Sipilä et al., 2010; Z. B. Wang et al., 2011), oxygenated organic compounds (Metzger et al., 2010; Riccobono et al., 2012; Riipinen et al., 2011; Tröstl et al., 2016; Y. Wang et al., 2015), ammonia and amines (Berndt et al., 2010; Jen et al., 2014; Kirkby et al., 2011; M. Wang et al., 2020; Yao et al., 2018), and iodine species (O’Dowd et al., 2002; Sipilä et al., 2016). However, there is a lack of detailed understanding of NPF under distinct environmental conditions with the contribution of multiple chemical components. NPF occurs less frequently in pristine conditions and more often in polluted environments, which contradicts the assumptions of low condensation sinks (Kulmala et al., 2017). This calls for focused observations of aerosols, precursor species, and meteorological variables in polluted and urban environments.

The Mediterranean region is a “hot spot” for climate change and is projected to undergo significant warming and drying in the 21st century (Tuel & Eltahir, 2020; Zittis et al., 2022). Continental, marine, and desert‐dust sources influence airmasses and intense photochemistry during the dry and hot weather makes this region particularly interesting for NPF (Lelieveld et al., 2016). NPF studies over the Mediterranean region are mainly from the northwestern basin (Berland et al., 2017; Brines et al., 2015; Casquero‐Vera et al., 2020; Hamed et al., 2007; Petäjä et al., 2007; Rose et al., 2015).

Investigations of NPF across the eastern Mediterranean region remain limited and present substantial gaps in field observations and relevant scientific understanding. Among the available NPF studies over the eastern Mediterranean, Kalivitis et al. (2019) reported the longest record (10 years), with NPF occurring on 27% of the available measurement days. Model simulations suggested that monoterpenes contribute to a large fraction of the observed NPF events. Pikridas et al. (2012) showed that the lack of ammonia may limit nucleation in sulfate‐rich environments, which is expected over the eastern Mediterranean. Baalbaki et al. (2021) showed that most NPF occurs during the daytime with few cases of nighttime NPF and high desert dust loading events, and they also reported that the particle formation rate and growth rate are comparable to those in urban environments. Berland et al. (2017) investigated daily horizontal and vertical development of nucleation phenomena. They suggested that NPF may have a spatial scale of several hundred kilometers, determined by synoptic conditions. Recently, Aktypis et al. (2024) examined the frequency and spatial extent (from 11 different locations in Greece) of the NPF events over the eastern Mediterranean during the summers of 2020 and 2021. These authors reported that the NPF frequency varied from 0% to 60% in the southwest to northern, central and eastern parts of the country and the high NPF frequency sites were linked to coal‐fired power plants with high sulfur dioxide emissions and agricultural areas with higher ammonia emissions. Kalivitis et al. (2015) studied the relationship between cloud condensation nuclei (CCN) and NPF; it was observed that smaller particles during NPF events are rich in organics and are less hygroscopic. However, another study from the same location found that although the CCN concentrations may be enhanced during the NPF events, the impact on cloud droplet formation is limited by the availability of water vapor (Kalkavouras et al., 2017).

This study presents the results from concurrent ambient measurements of particle number size distributions (PNSDs), non‐refractory sub‐micron (NR‐PM_1_) aerosol chemical composition, and trace gases conducted at an urban site in the eastern Mediterranean, at the city of Rehovot, Israel. Our study is focused on secondary aerosols. The objectives of the study are (a) to characterize the NPF events over the area of the study, (b) to identify chemical components relevant to secondary aerosol formation and growth, and (c) to understand the relationship between prevailing meteorology and NPF events.

This study employs hybrid positive matrix factorization (PMF) analysis to address these objectives, a novel approach to understanding the relationship between particle size distribution and chemical composition (Nursanto et al., 2023). The hybrid PMF approach integrates the data sets obtained from the high‐resolution time‐of‐flight aerosol mass spectrometer (HR‐ToF AMS) and aerosol number size distributions from the scanning mobility particle sizer (SMPS) into the input of the hybrid PMF analysis. Further, the aerosol characteristics during two nationwide biomass‐burning events (2021 and 2023) are also presented to assess the impact of biomass‐burning events on NPF and aerosol growth.

## 2 Methods and Materials

### 2.1 Sampling Site and Meteorological Conditions

Aerosol and trace gas measurements were conducted during two intensive campaigns from 29 April to 3 May 2021 (Campaign 1) and from 3 May to 11 May 2023 (Campaign 2) at the Department of Earth and Planetary Sciences, Weizmann Institute of Science in Rehovot, Israel (31°53″N 34°48″E). Rehovot is an urban area surrounded by small cities and agricultural settlements 15 km from the Mediterranean Sea. Ambient air was sampled through a 3/8″ conducting tubing with a PM_10_ inlet from the rooftop to the instrument (∼80 m AMSL). More details on the observational site and general features of the meteorology are available elsewhere (Adler et al., 2011; Tomlin et al., 2022). Figure S1 in Supporting Information S1 shows the measurement location and the isentropic 5‐day air mass back‐trajectories, calculated using the Hybrid Single‐Particle Lagrangian Integrated Trajectory, that arrived at the measurement site during the campaigns. Analysis of air mass trajectories indicated that on most days, the air mass arrived from adjacent continental regions, mostly bypassing the marine regions. In contrast, the air mass originated from the Mediterranean Sea on 30 April 2021, 06 May 2023, and 12 May 2023, suggesting a marine influence.

The details regarding the meteorological conditions during the observation period are listed in Table S1 in Supporting Information S1. The mean values for the air temperature, RH, and wind speed were 22.9 ± 4.9°C and 21.1 ± 4.7°C, 55.5 ± 19.1% and 64.6 ± 17.5%, 2.20 ± 1.2 m s^−1^ and 2.0 ± 1.3 ms^−1^, respectively during Campaign 1 and Campaign 2.

### 2.2 Instrumentation

The particle size distributions in the size range of 15.1–662 nm were measured using a SMPS at 5‐min intervals. The SMPS system consists of a differential mobility analyzer (DMA, TSI 3081) with an X‐ray neutralizer and a butanol‐based condensation particle counter (CPC, TSI 3776). The SMPS in this study was operated with a sheath and sample flow rates of 3 L min^−1^ and 0.3 L min^−1^, respectively. All recommended multi‐charge and diffusion corrections were applied using the manufacturer's software and algorithms (Aerosol Instrument Manager 9.0.0.0). The minimum detection size in the present study is 15.1 nm. Therefore, any insights into particles below this threshold are not encompassed in this study, and as such, the lowest bin does not indicate nascent particles. The SMPS measurements indicate particles that have been grown after the gas‐to‐particle conversion. For readability, in this study, we refer to the secondary aerosol formation process as NPF events, and the term ultrafine particle burst events (Kompalli et al., 2020; Yadav et al., 2021) is not used.

The mass concentrations of non‐refractory PM_1_ (NR‐ PM_1_) species (including organics, sulfate, nitrate, ammonium, and chloride) were measured using a high‐resolution time‐of‐flight aerosol mass spectrometer (HR‐ToF‐AMS). The working principle of AMS and its modes of operation are detailed elsewhere (Canagaratna et al., 2007; Drewnick et al., 2005; Jayne et al., 2000). The AMS was operated alternately in V‐mode (standard reflectron‐ ToF mass spectrometer configuration) and W‐mode (with an additional hard mirror for high‐resolution measurements) during Campaign 2, each with a 1‐min sampling time, and only in V‐mode during Campaign 1 with a 3‐min sampling time. Inlet sample flow, particle sizing, and ionization efficiencies were calibrated before each campaign following the standard protocols (Jimenez et al., 2003). All the collected data has been processed using the software provided by the manufacturer (Squirrel version 1.65 and PIKA version 1.25 within IGOR Pro version 6.3.7.2). Since the AMS used in this study consists of a standard vapourizer, a composition‐dependent collection efficiency was applied following Middlebrook et al. (2012).

Collocated real‐time measurements of trace gas concentrations and meteorological parameters [temperature (T), RH, and solar radiation] were also used to support the aerosol chemical composition measurements. An online ultraviolet photometric ozone analyzer (model: 49i, Thermo Scientific, USA), chemiluminescence NO_x_ analyzer (Model: 405 nm, 2B Technologies, USA) and a pulsed fluorescence SO_2_ monitor (Model 43i, Thermo Scientific, USA) were used to measure the surface level concentration of ozone, NO_x_, and sulfur dioxide respectively. The measurements of meteorological parameters were obtained from a weather station located 1 km to the east of the measurement site.

During Campaign 2, atmospheric particles were collected during selected time periods through impaction using a rotating ten‐stage Micro‐Orifice Uniform Deposit Impactor (MOUDI 110‐R, MSP Inc). Samples for analysis were chosen from stage 10 with cut‐off aerodynamic diameter of *D*_*50*_ = 56 nm. Individual particles were also sampled via impaction onto plasma‐cleaned silicon wafers (Silson Ltd.), and Carbon type‐B thin film on Cu 400 mesh TEM grids (Ted Pella, Inc.). Following collection, these particle‐loaded substrates were placed into plastic containers, sealed with Parafilm™, and stored in desiccator cabinets prior to analysis.

The chemical imaging of individual particles collected by the MOUDI impactor was performed using scanning transmission electron microscopy (STXM) coupled to near‐edge X‐ray absorption fine structure (NEXAFS) spectroscopy, located at a beamline 5.3.2.2 within the Advanced Light Source synchrotron facility at the Lawrence Berkeley National Laboratory, Berkeley, CA. Operational specifics of the instrument are detailed in Kilcoyne et al. (2003). For this work, all measurements were conducted at the carbon K‐edge energy range (278–320 eV) to explore the chemical bonding of carbon within individual particles. This enabled quantifying crucial properties of atmospheric aerosols, including organic carbon (OC) speciation, organic volume fraction (OVF), and chemical mixing state information, employing methodologies summarized in the Texts S1–S4 in Supporting Information S1 (Fraund et al., 2019; Moffet et al., 2016; O’Brien et al., 2015; Rivera‐Adorno et al., 2024; Signorell & Reid, 2010; Tomlin et al., 2022).

### 2.3 Condensation Sink Calculations

The condensation sink (CS) represents the loss rate for the condensable vapors due to condensation on the pre‐existing particles (Kulmala et al., 2001). Following the methodology provided by Kulmala et al. (2001), the CS can be calculated as (Kompalli et al., 2020; Yadav et al., 2021),

(1) $$\text{CS}=2\pi D\sum_{i}\beta_{i}d_{pi}N_{i}$$

where

(2) $$\beta_{i}=\frac{1+K_{n}}{1+\left(\frac{4}{3\alpha_{m}+0.337}\right)K_{n}+\frac{4}{3\alpha_{m}}K_{n}^{2}}$$

D—diffusion coefficient of the condensable vapor,

$\beta_{i}$—size‐dependent transition correction factor,

$d_{pi}$—diameter of the particle in bin *i* (nm),

$N_{i}$—particle number concentration in bin *i* (cm^−3^),

$\alpha_{i}$—sticking coefficient (assumed to be unity),

$K_{n}$—Knudsen number.

### 2.4 Sulfuric Acid Proxy Calculations

Sulfuric acid is a major driver of NPF (Kerminen et al., 2018; Kulmala, Kerminen, et al., 2004). Direct measurements of sulfuric acid are challenging, and proxies for sulfuric acid are usually derived from ancillary data (Dada et al., 2020; Lu et al., 2019; Petäjä et al., 2009). Here, the proxy for sulfuric acid concentration was calculated following Mikkonen parametrization (Mikkonen et al., 2011).

(3) $$\left[\text{Sulfuric}\;\text{acid}\right]=8.21\times 10^{-3}\times kR\times {\left[SO_{2}\right]}^{0.62}{\times \left[\text{CS}\times \text{RH}\right]}^{-0.13}$$

[Sulfuric acid]—concentration of gaseous sulfuric acid (molecules cm^−3^), *k*—reaction coefficient for the reaction between SO_2_ and OH‧ (molecules^−1^ s^−1^ cm^3^) = 1.03495,

*R*—solar flux (W m^−2^),

[SO_2_]—concentration of sulfur dioxide (ppb),

CS—condensation sink (s^−1^),

RH—relative humidity (%).

In situ measurements of SO_2_, RH, solar radiation and derived values of CS were used in this study to estimate the sulfuric acid proxy concentrations.

### 2.5 Classification of the NPF Events

We applied the criteria outlined by Dal Maso et al. (2005) to categorize NPF events. Specifically, to qualify as an NPF event, the following conditions were required: (a) emergence of a distinct mode of particles in the number size distributions in the nucleation range, (b) predominance of this new mode for a duration surpassing 1 hour, and (c) observable growth of particles into larger size ranges. Furthermore, the observed NPF events were subdivided into types 1a, 1b, and 2 based on the criteria described in the previous studies (Dal Maso et al., 2005; Hirsikko et al., 2007; Yli‐Juuti et al., 2009). Type 1a is a nucleation event with a well‐defined continuous growth to a larger size for several hours. Type 1b is a similar nucleation event, but the growth curve does not exhibit a well‐defined shape. Type 2 is a burst of ultrafine mode particles without any discernible growth.

### 2.6 Source Apportionment of Organic Aerosols

PMF analysis was performed to decipher the high‐resolution AMS data sets, utilizing PMF2 executable version 4.2 in robust mode and PMF evaluation tool (PET) version 2.3 (Ulbrich et al., 2009). Standard pre‐treatments of the PMF input data matrix were applied, including removing “bad data” with low signal‐to‐noise ratio (SNR < 0.2) and down‐weighing weak signals (0.2 < SNR < 2), as suggested by Ulbrich et al. (2009). The number of factors was carefully chosen by running PMF with five factors and changing the rotational forcing parameter (“fpeak”) incrementally by 0.1, based on the minimum Q value (which is the “PMF quality‐of‐fit parameter”), the correlation between the factors (mass spectra and time series), and comparison with the AMS database (Zhang et al., 2011).

PMF analysis on the AMS data sets yielded a 4‐factor solution during Campaign 1 and a 5‐factor solution during Campaign 2. The mass spectrometry profiles for the respective factors are shown in Figures S2 and S3 in Supporting Information S1. Two factors were primary emitted organic aerosol components, namely– HOA (hydrocarbon‐like organic aerosol) and BBOA (biomass‐burning organic aerosols), and two other components were related to secondary aerosols‐namely more‐oxidized oxygenated organic aerosol (MO‐OOA) and less‐oxidized oxygenated organic aerosol (LO‐OOA). The BBOA factor was further separated into LO‐BBOA (low‐oxidized BBOA) and MO‐BBOA (more‐oxidized BBOA) during Campaign 2. HOA, a surrogate of combustion‐generated organic aerosols, was selected based on the presence of saturated (C_n_H_2n+1_, peaks at m/z 29, 43, 57 and 71) and unsaturated (C_n_H_2n−1_, peaks at m/z 27, 41, 55, 69, and 83) hydrocarbon signatures (Aiken et al., 2007). OOA represents oxidized organic aerosols and was separated based on the strength of the *f*_*44*_ signal. The dominant peaks at *m/z* 43 and *m/z* 44 were used to further separate OOA into two sub‐components, LO‐OOA (with a lower *f*_*44*_ to *f*_*43*_ ratio suggesting fresh particles) and MO‐OOA (with a higher *f*_*44*_ to *f*_*43*_ ratio suggesting aged particle) (Jimenez et al., 2009). The BBOA factor was distinguished by prominent signals at m/z 29, 60, and 73 representing the anhydrous sugars like levoglucosan and manosan (dominant during wood burning) (Aiken et al., 2007). Further, the BBOA was separated into MO‐BBOA (with a higher *f*_*44*_ to *f*_*43*_ ratio suggesting aged particles) and LO‐BBOA (with a lower *f*_*44*_ to *f*_*43*_ ratio suggesting fresh particles). BBOA factor was observed only during and after the bonfire Lag BaO'mer festival, indicating the absence of wood‐burning/biomass‐burning sources in the region on the other sampling days.

## 3 Results and Discussion

### 3.1 General Overview of the Aerosol Characteristics and NPF Events

**Figure 1:**
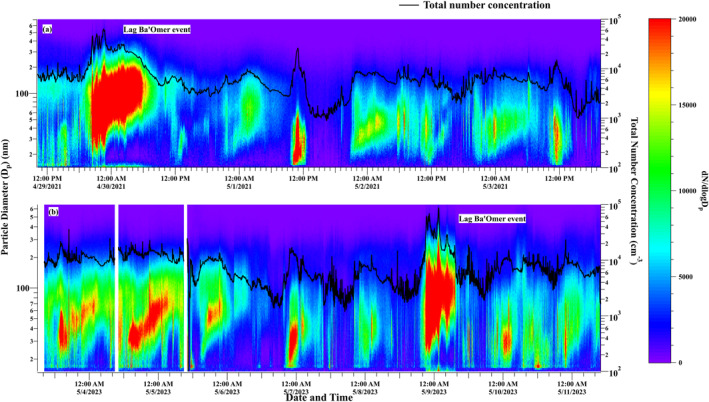
Temporal variation of aerosol number size distribution, total number concentration (black line and right *y*‐axis) for the measurement periods during the measurement campaigns conducted in panels (a) 2021 and (b) 2023. The color bar indicates dN/dlogDp. The date and time are given in local time (UTC + 3).

The temporal variation of PNSD and total number concentration during (a) Campaign 1 (29 April—03 May 2021) and (b) Campaign 2 (3 April—11 May 2023) are shown in Figure 1. Notable enhancement in the aerosol number concentration (>60,000 cm^−3^) and particle size were observed during the national Lag Ba’Omer bonfire festival (marked in Figure 1), which is comparable to the previous measurements from the same site in 2018 during the festival period (Tomlin et al., 2022). Frequent NPF events with increased number concentrations in the lowest bin and subsequent growth to larger sizes have been detected. A total of 16 NPF events were observed during the two campaigns, in both daytime and nighttime. Interestingly, NPF occurred on all the days of the campaign period. A long‐term study, 1‐year of continuous measurements by Baalbaki et al. (2021) reported high frequency (56.7%) of days with NPF events in Cyprus in the eastern Mediterranean region. Similarly, Debevec et al. (2018) also reported a high frequency of NPF events in Cyprus (14 out of 20 observation days, 70%). In contrast, lower frequency of occurrence (27% of the available days) in 10 years of observation (Kalivitis et al., 2019) and an annual frequency of 36% (Berland et al., 2017) of NPF events were reported from Crete, Greece. Although the observation period in the present study is shorter than the other reported studies from the Eastern Mediterranean, the high frequency of occurrence of the NPF events during both day and night periods is unique in this study location.

Prior to the events, the background values for geometric mean diameter (GMD) and total particle number concentrations (mean values of 2‐to‐3 hours before the events) were 61.4 ± 4.9 nm and 4,500 ± 1,800 cm^−3^ during Campaign‐1 and 66.5 ± 12.7 nm and 6,600 ± 3,900 cm^−3^ during Campaign‐2, respectively. Table 1 lists the mean value of major NPF‐relevant parameters measured during the present study. The aerosol number concentration was 5,400 ± 2700 cm^−3^, with a GMD value of 58.1 ± 10 nm during Campaign 1 and 7,300 ± 4,000 cm^−3^, with a GMD value of 61.4 ± 11.5 nm during Campaign 2. The ozone concentration remained comparable during the two campaigns (31 ± 15 and 34 ± 17 ppb during 2021 and 2023, respectively), and SO_2_ showed higher concentrations during Campaign 1 (0.43 ± 0.44 ppb) compared to Campaign 2 (0.09 ± 0.11 ppb). The mean NR‐PM1 chemical composition showed that organics contributed the major component [mass fraction (MF) ∼ 0.52 and 0.57 during Campaign 1 and 2, respectively], followed by sulfate (MF ∼ 0.28 and 0.24 during 2021 and 2023, respectively), ammonium (MF∼ 0.11 and 0.11 during 2021 and 2023, respectively) and nitrate (MF∼ 0.07 during 2021 and 2023).

**Table 1:** Average Values for Various Aerosol and Trace Gas Parameters During the Measurement Campaigns in 2021 and 2023

	During Lag Ba’Omer event		Excluding Lag Ba’Omer event	
	Campaign 1	Campaign 2	Campaign 1	Campaign 2
Total Number concentration (cm^−3^)	19,000 ± 12,700	26,300 ± 16,500	5,400 ± 2700	7,300 ± 4,000
GMD (nm)	84.7 ± 13.6	79.1 ± 11.7	58.2 ± 10.0	61.4 ± 11.5
Ozone (ppb)	29.2 ± 8.8	17.5 ± 7.5	31.3 ± 15.7	34.2 ± 16.7
NO_x_		18.0 ± 5.1		14.6 ± 9
SO_2_ (ppb)	0.67 ± 0.62	0.15 ± 0.24	0.43 ± 0.44	0.09 ± 0.11
Organics mass fraction	0.57	0.77	0.52	0.57
Sulfate mass fraction	0.22	0.07	0.28	0.24
Ammonium mass fraction	0.10	0.05	0.11	0.11
Nitrate mass fraction	0.09	0.08	0.07	0.07
Chloride mass fraction	0.02	0.03	0.02	0.01

During the Lag Ba’Omer bonfire event, the aerosol number concentrations increased almost 4‐fold, reaching 19,000–26,000 cm^−3^, and GMD increased to 79–84 nm. Notably, except during the Lag Ba’Omer event, the GMD values were mostly below 70 nm. The mass fractions of the organics (MF ∼ 0.57 during Campaign 2) and sulfate (MF ∼ 0.22 during Campaign 2) remained almost the same during the Lag Ba’Omer event compared to the background conditions.

### 3.2 The Driving Parameters for NPF Events

**Figure 2:**
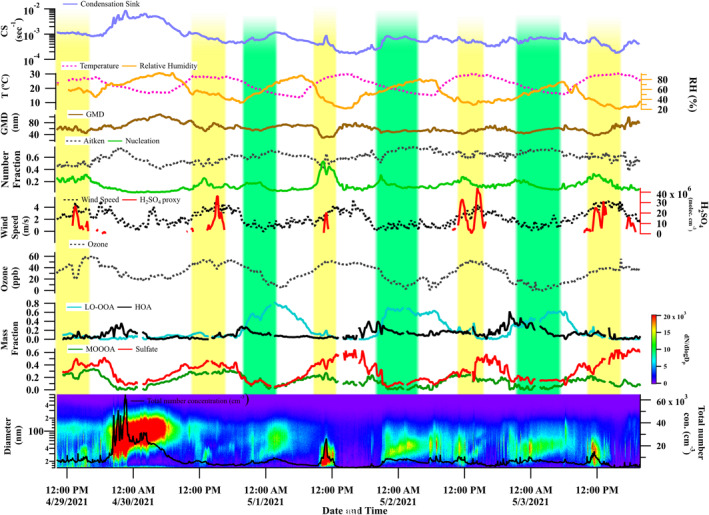
Temporal variation of (a) condensation sink, (b) temperature, relative humidity, (c) geometric mean diameter, (d) number fraction of nucleation and Aitken particles, (e) wind speed, concentration of sulfuric acid proxy, (f) concentration of ozone, mass fraction (in the NR‐PM_1_ mass loading) of (g) hydrocarbon like organic aerosols (OA), low‐oxidized oxygenated OA and (h) more‐oxidized oxygenated OA, sulfate and (i) total number concentration during Campaign 1. Daytime and nighttime events are highlighted in yellow and green respectively.

**Figure 3:**
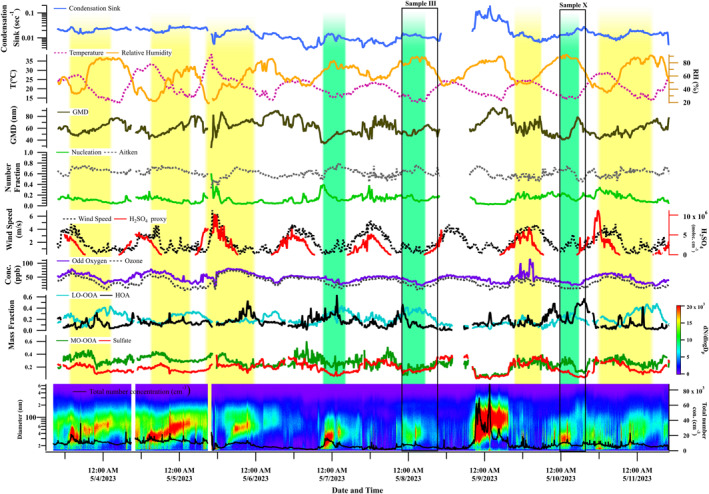
Temporal variation of (a) condensation sink, (b) temperature, relative humidity, (c) geometric mean diameter GMD, (d) number fraction of nucleation and Aitken particles, (e) wind speed, concentration of sulfuric acid proxy, (f) concentration of ozone, mass fraction (in the NR‐PM_1_ mass loading) of (g) hydrocarbon like organic aerosols (OA), low‐oxidized oxygenated OA and (h) more‐oxidized oxygenated OA, sulfate and (i) total number concentrations during Campaign 2. Daytime and nighttime events are highlighted in yellow and green, respectively.

The temporal variation of the meteorological parameters, GMD, aerosol nucleation and Aitken mode fraction (in the total particle concentrations), sulfuric acid‐proxy values, ozone/odd oxygen, and MF of PMF‐derived components are shown in Figure 2 for Campaign 1 and in Figure 3 for Campaign 2. The NPF events are highlighted in the figures. Daytime events are marked by yellow color, and nighttime events are marked by green color. An increase in the fraction of nucleation‐mode particles (>0.10 and hence a decrease in the Aitken‐mode aerosol fraction) with a lower GMD value (<50 nm) is considered as an NPF event. As the fraction of nucleation particles decreases, simultaneous enhancements in the GMD are observed following the events.

The mean CS values were (9.4 ± 4.0) × 10^−3^ s^−1^ during Campaign 1 and (14.2 ± 6) × 10^−3^ s^−1^ during Campaign 2, excluding the Lag Ba’Omer event. These CS values were comparable to the annual average values reported from the eastern Mediterranean (14.0 ± 15.9) × 10^−3^ s^−1^ (Pikridas et al., 2012), and (8.9 ± 3.3) × 10^−3^ s^−1^ (Hussein et al., 2019). The CS, which is significantly influenced by the background aerosol number‐size distribution, represents the rate at which low‐volatility vapors condense onto existing aerosol particles, thereby inhibiting the nucleation of new particles. However, this study found no clear correlation between CS values and NPF events.

It is clear from both figures that an enhancement in the concentrations of sulfuric acid proxy is concurrent with all daytime events. The average concentration of sulfuric acid proxy showed an order of magnitude difference between Campaign 1 [mean ∼ (12.0 ± 31.0) × 10^6^ molecules cm^−3^] and Campaign 2 [mean ∼ (2.64 ± 2.1) × 10^6^ molecules cm^−3^]. Along with sulfuric acid proxy, higher odd oxygen/ozone levels were observed in all daytime events. Although a direct association between the events and odd oxygen cannot be established, elevated levels of odd oxygen can indicate higher atmospheric oxidative capacity and, hence, photochemical formation of secondary species favoring particle growth (Ajith et al., 2023; Clapp & Jenkin, 2001; Wood et al., 2010). The simultaneous occurrence of higher levels of sulfuric acid proxy and odd oxygen during daytime events suggests the possible photochemical formation of sulfuric acid and secondary species responsible for the nucleation and growth. The contribution of sulfuric acid to daytime NPF is not surprising since it is the major species responsible for NPF under atmospheric conditions. Recent studies showed that, along with sulfuric acid, different compounds help stabilize the initial cluster formations and participate in the NPF. These include monoterpenes, dimethylamines (Kirkby et al., 2023; Yao et al., 2018), and low‐volatility organics vapors (Tröstl et al., 2016).

The sulfuric acid proxy concentration in this study falls within the reported range (3 × 10^5^–1 × 10^7^ molecules cm^−3^) from Cyprus (Baalbaki et al., 2021). The study by Baalbaki et al. (2021) reported a relationship between the particle formation rate (J_1.5_) and the sulfuric acid proxy that was highly variable throughout the year. The presence of stabilizing compounds was used to explain the higher formation rate during winter, even in events with lower sulfuric acid concentrations. A highly varying value for the sulfuric acid proxy was reported from another eastern Mediterranean location—Crete, during the FAME‐08 (0.5–2.0 × 10^6^ molecules cm^−3^, early summer) and FAME‐09 (∼10^7^ molecules cm^−3^, late winter) campaigns by Pikridas et al. (2012). They attributed the observed lower nucleation rate during summer to the acidic nature of the aerosols and the lack of gas‐phase ammonia/amines. However, in our study, the aerosols were completely neutralized. The corresponding aerosol neutralization ratio, the ratio between measured ammonium and the total ammonium required to neutralize the major anions (Zhang et al., 2007) had a value of 0.98 ± 0.25 during Campaign 2 and 0.90 ± 0.14 in Campaign 1, excluding the bonfire event.

The ambient temperatures in our study consistently exceeded 26°C, with an approximate RH below 40% during the daytime events. Hot and dry conditions are typically favorable for NPF (Dada et al., 2017; Kanawade et al., 2014; Pushpawela et al., 2018; Salma et al., 2016). Interestingly, higher wind speeds (∼4 ms^−1^ during both campaign periods) persisted during all daytime events. High wind speed may cause the dispersion of the pre‐existing particles and the reduction in CS, which also favors NPF (Hama et al., 2017). However, a clear dependence of NPF on the wind speed could not be established due to covariation of multiple parameters. The MF of the secondary species, such as sulfate and MO‐OOA, was also enhanced during the daytime events. Overall, several parameters, such as the concentration of sulfuric acid proxy, odd oxygen, wind speeds, and the MF of major secondary species, increased during the daytime events, highlighting their possible contributions to the events. Due to the complexity of NPF events, their growth mechanism, and the involvement of multiple components, distinguishing the contribution of each parameter is challenging. The identification of the probable species participating in NPF and their subsequent growth, along with the meteorological parameters required for these events, is discussed in Section 3.4. Specifically, we examine higher condensable vapor concentrations, lower CS values, and triggering atmospheric dynamical processes as key conditions for NPF events.

The nighttime events were associated with an increase in the nucleation‐mode fraction (>0.1) followed by growth to the Aitken‐mode size range. A notable feature of the nighttime events (unlike the daytime events) was the disturbance of particle growth by the sudden appearance of high concentrations of larger particles (∼100 nm). The estimated sulfuric acid proxy values were zero during nighttime events due to the absence of solar radiation. In contrast to the daytime conditions, lower temperatures (<20°C) and calmer conditions persisted during nighttime. The MF of sulfate, MO‐OOA, and HOA slightly changed during the initial stages of the events. A higher LO‐OOA fraction (>0.30 during Campaign 2 and >0.40 during Campaign 1) was observed during nighttime, suggesting the probable species responsible for the growth. Overall, the nighttime events observed during this study consist of low temperatures, humid conditions, calm winds, and higher LO‐OOA.

Nighttime NPF events have also been reported in other eastern Mediterranean studies (Baalbaki et al., 2021; Kalivitis et al., 2019; Kopanakis et al., 2013; Pikridas et al., 2012). Kalivitis et al. (2012) reported a plausible association of nighttime ion concentrations with NPF. In their study, the nighttime events were more frequent during spring and autumn and when air masses traveled over the islands, indicating the contribution of local biogenic sources. Kalivitis et al. (2019) also reported nighttime events from the same station. The events had no or slow growth rate due to the limited availability of condensable vapors, and the sources were assumed to be local rather than regional. Recently, Baalbaki et al. (2021) observed high cluster mode concentrations during nighttime associated with episodes of high desert dust loadings in Cyprus. However, all these studies are reported from rural sites, unlike the urban location in the present study, and hence, a similar NPF mechanism is not to be expected.

Table S2 in Supporting Information S1 lists the mean meteorological and aerosol parameters separated for daytime and nighttime events. In summary, daytime events consist of hot and dry conditions (T ∼ 24.7 ± 4.9°C and RH ∼ 47.6 ± 18.6%) in contrast to the cooler and humid conditions during nighttime events (T ∼ 17.0 ± 2.2°C and RH ∼ 70.5 ± 11.5%). Further, daytime events were aided by more inorganic fraction (MF of sulfate ∼ 0.23) due to photochemical reactions. In contrast, nighttime events consist of more organic compounds (MF of LO‐OOA ∼ 0.41) due to their less photochemical degradation and/or direct sources. Thus, the meteorological conditions and the fraction of inorganic and organic species are different during daytime and nighttime events, suggesting different formation/growth mechanisms.

### 3.3 Events of Intense Biomass Burning Emissions

**Figure 4:**
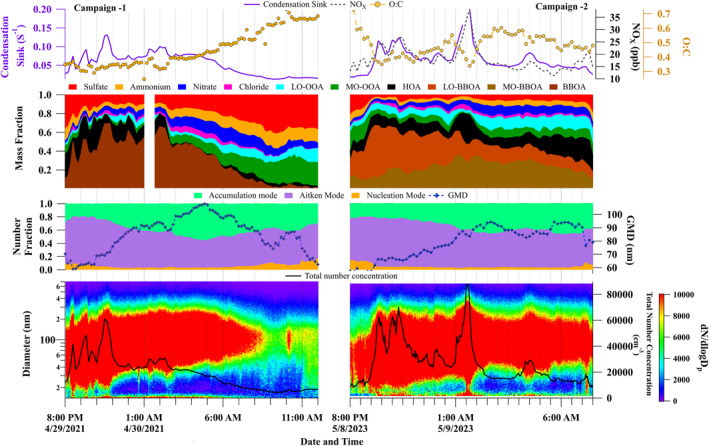
Temporal evolution of Condensation sink, NOx, O:C, mass fraction (in the NR‐PM1 mass loading) of inorganic and organic species, number fraction of nucleation, Aitken, and accumulation mode particle, geometric mean diameter, number size distribution, and total number concentration during Campaign 1 and 2.

The observation period covered local anthropogenic biomass burning events related to a nationwide bonfire event, celebrated with night‐time bonfires, on 8–9 May 2023 and 29–30 April 2021. The fires start around sunset (∼19:30 Local Time) and end by sunrise. Figure 4 shows the time series of aerosol number size distribution, total number concentration, GMD, fraction of nucleation, Aitken and accumulation mode particles, MF of inorganics and organics, CS, NO_x_, and O:C ratio of bulk organics during the Lag BaO'mer events.

An enhancement in the fine mode [fraction of (nucleation + Aitken) mode aerosols >0.8] and lower GMD values (∼60 nm) were observed during the onset of the bonfire (∼20:00 local time during 2021 and 2023). A clear enhancement in nucleation mode (fraction > 0.1) was observed at 20:00 local time, indicating the initiation of the event. It is interesting to notice a significant amount of nucleation mode particles despite a strong reservoir of larger particles during these episodes, highlighting that multiple atmospheric processes are acting in tandem. The fractional contributions of various factors in the PMF‐derived factors indicated a clear presence of fresh BBOA (BBOA during 2021 and LO‐BBOA during 2023), which depicted a higher mass fraction (MF ∼ 0.20–0.40) during the onset of the bonfire event. Further, the lower O:C ratios (∼0.30–0.40) during the onset indicated that this fraction of particles is less oxidized and freshly emitted. After 20:00 local time, the GMD values increased steadily with a simultaneous rise in accumulation mode particle concentrations, indicating the growth of newly formed/freshly emitted particles to larger sizes, which is also reflected in the sudden change in CS values (>0.05). During Campaign 2, the MF of LO‐OOA (indicating fresh BBOA) decreased and MO‐BBOA increased, suggesting the conversion of LO‐BBOA to MO‐BBOA along with the change in GMD. Supporting this, the O:C ratio also increased from 0.4 to 0.6 during 2023 and 0.3 to 0.7 in 2021. Even though the CS values are high, several NPF events were observed, possibly due to the abundance of precursors (Kanawade et al., 2022; Kompalli et al., 2018; Yadav et al., 2021) that overcome the barrier created by the higher CS. However, these bursts persisted for a few minutes and merged with the large sink of pre‐existing particles. The NPF events observed in the Lag Ba’Omer event were mainly triggered by the large amounts of anthropogenic biomass burning emanated vapors, which overcame the barrier created by the simultaneous presence of pre‐existing particles that acted as a sink for these bursts and suppressed further nucleation and rapid growth.

The most important observation during the Lag Ba’Omer events is a large influx of Aitken mode particles during intense biomass burning periods, which subsequently evolve toward the accumulation mode with a change in the overall aerosol oxidation degree. These biomass‐burning episodes emit significant amounts of condensable vapors and primary particles. Such emissions undergo various atmospheric processes, including steady coagulation (which is particle size‐dependent), condensation of the low‐volatile vapors on the pre‐existing particles, and the possible nighttime time oxidative aging by nitrate radical and ozone might have contributed to the particle evolution and hence the increase in O:C. Interestingly, during Campaign 2, the oxidative aging of the particles continued the next day, possibly due to the photochemical processing as seen in the further increase in the O:C ratio.

Recently, Tomlin et al. (2022) investigated the physical and chemical transformation of the biomass burning aerosols during the Lag Ba’Omer 2018 event sampled at the same site. They observed the higher mixing of the particles with an increase in particle organic fraction suggesting the aging of the emitted particles after the event. Adler et al. (2011) also reported an evolution of biomass‐burning aerosols during the Lag Ba’Omer 2009 event, with a shift in modal diameter, oxidation degree, and higher organic mass concentration observed following the biomass‐burning event. Adler et al. (2011) reported a higher *f*_*44*_ *to f*_*43*_ (fractional contribution of ion signal at 44 and 43 m/z) ratio and relatively low values of *f*_*57*_ (∼0.02), indicating the formation of more semi‐volatile oxygenated organic aerosols. Notably, their study showed an increase in secondary organic aerosol mass concentration on the day following the event, attributed to the photochemical oxidation of volatile organics by ozone and OH.

### 3.4 Chemical Characterization of NPF Events

#### 3.4.1 Hybrid PMF Analysis

A hybrid PMF analysis was employed to understand the chemical species responsible for the NPF events and growth. This approach involved combining the aerosol chemical composition data obtained from AMS with the concurrent PNSD measurements, a method known as the “hybrid PMF analysis” (Nursanto et al., 2023). A detailed procedure for performing the hybrid PMF analysis, along with the criteria for factor selection, is provided in the Text S5 in Supporting Information S1. Briefly, the analysis combined the 10‐min average of organic mass spectra in the m/z range of 12–120 with inorganic mass concentrations (sulfate, ammonium, nitrate, and chloride), as well as the 10‐min averaged particle number concentrations (*dN*) across 28 diameter size bins, derived from a total of 105 size bins of the SMPS. These combined data matrices were used to generate the input for the hybrid PMF analysis. Since the inorganic mass concentrations and number size distributions exhibit a different magnitude in comparison to the organic data matrix, a downweighing constant is applied to align their magnitudes. The errors for the organics and inorganics were generated using the standard AMS analysis software (Squirrel), and the errors for the size bins are the standard deviation of the raw data (standard deviation obtained while averaging the 3‐min data to 10 min). The PMF analysis was performed using the PMF evaluation tool PET v3.08 (Ulbrich et al., 2009). The key benefit of performing hybrid PMF analysis is the additional information regarding inorganics and size contribution apart from organics in the factor profiles.

Since the events observed during Campaign 1 consisted of mostly disturbed events with simultaneous emissions in the larger and smaller size range (as shown in Figure 1), it is difficult to assign the size‐segregated factors during the analysis. Hence, physically meaningful solutions were not obtained while performing hybrid PMF analysis and were not considered for interpretation. Because the daytime and nighttime NPF events have different formation/growth mechanisms, this study conducts the hybrid PMF analysis separately for daytime and nighttime NPF events observed during Campaign 2. Three daytime events (3–6 May 2023) and three nighttime events (6–10 May 2023) during Campaign 2 were considered.

**Figure 5:**
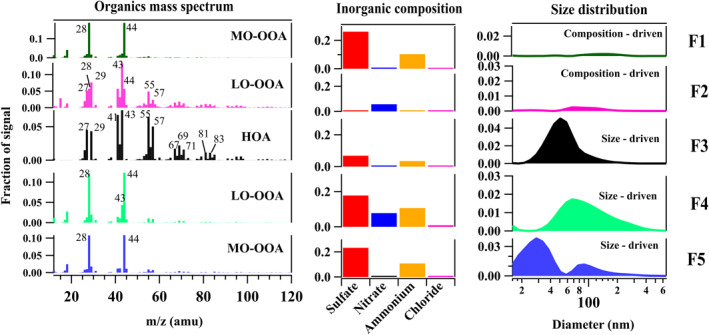
Hybrid positive matrix factorization derived factors (F1–F5) obtained for daytime New particle formation events during Campaign 2. Each factor is split into 3 panels with separate axis. The panels on the left show the organic mass spectrum, the panels on the middle show the inorganic mass concentration (sulfate, nitrate, ammonium, chloride), and the panels on the right show the particle number size distribution (*x*‐axis (size) in logarithmic scale). The factors are assigned as (F1) composition‐driven sulfate + ammonium + more‐oxidized OOA (MO‐OOA), (F2) composition‐driven nitrate + less‐oxidized OOA (LO‐OOA), (F3) size‐driven sulfate + ammonium + hydrocarbon like OA (HOA), (F4) size‐driven sulfate + ammonium + nitrate + LO‐OOA, (F5) size‐driven sulfate + ammonium + MO‐OOA.

Figure 5 depicts results of the hybrid PMF analysis for the daytime events. Each factor is divided into three panels: organic mass spectrum, inorganic mass concentration, and PNSD. A five‐factor solution comprised two composition‐driven factors (F1 and F2) and three size‐driven factors (F3, F4, and F5). The inorganic signals in the derived hybrid PMF factors were upweighed (multiplying with the downweighing constant), and the MF of each aerosol species (excluding the contribution from size distribution) is listed in Table S3 in Supporting Information S1.

The size‐driven factor‐5 (Figure 5 and Table S3 in Supporting Information S1) includes nucleation and the Aitken mode particles, indicating the initiation/occurrence of the NPF event. Factor‐5 consists of MO‐OOA (MF ∼ (0.42), sulfate (MF ∼ 0.40)), and ammonium (MF ∼ 0.18) as major components. The presence of ammonium along with sulfate suggests the neutralization of sulfate and the possible existence of ammonium sulfate. Two additional size‐driven factors (F3 and F4) were also obtained with a higher mode in the particle size distribution compared to factor 5. Factor‐4 consists of sulfate (MF ∼ 0.27), ammonium (MF ∼ 0.16) and HOA (MF ∼ 0.44) with a mode of ∼50 nm, whereas factor‐3 consists of a slightly higher mode of ∼70 nm with the composition of sulfate (MF ∼ 0.14), nitrate (MF ∼ 0.00), ammonium (MF ∼ 0.07) and LO‐OOA (MF ∼ 0.78).

**Figure 6:**
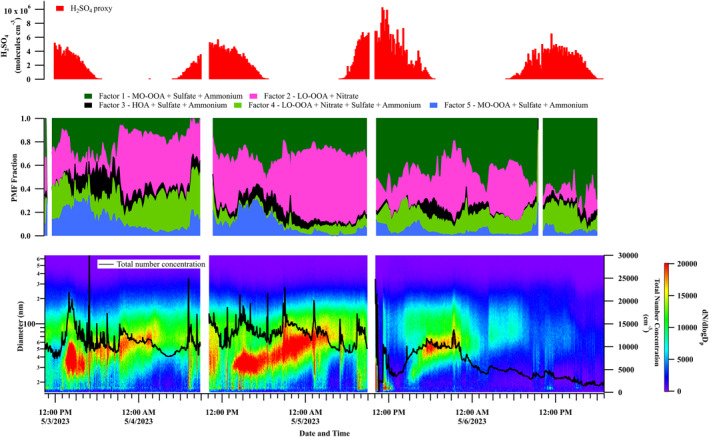
Time series of sulfuric acid proxy concentrations (in molecules cm^−3^), a fraction of hybrid positive matrix factorization derived factors for daytime events during Campaign 2, particle number size distributions and total number concentrations.

Figure 6 depicts the temporal variation of aerosol number size distributions, the fraction of hybrid PMF‐derived factors, and the concentration of sulfuric acid proxy during the selected daytime events. An enhancement in the size‐driven factor‐5 and sulfuric acid proxy is seen with the initiation of the NPF event. This strongly suggests the formation of ammonium sulfate from the reaction between sulfuric acid and ammonia and the uptake of low‐volatile organic compounds, contributing to the nucleation/Aitken mode in the PNSD. This is not surprising as several aerosol chamber‐based studies also reported the involvement of low‐volatility organic compounds and highly oxygenated organic molecules from the anthropogenic and biogenic sources in the nucleation and growth (Mohr et al., 2019; Riccobono et al., 2012; Tröstl et al., 2016).

Following the nucleation, the dominance of F5 decreased, and the contribution from F3 and F4 increased, indicating the growth of the nucleated particles to higher size ranges. The contribution of F3 is less but not negligible during the initial growth phase. The presence of F3 with a chemical makeup of HOA and ammonium sulfate suggests that the transported traffic emissions or the local organic emissions may contribute to this intermediate growth phase. Later, the growth is aided by the F4 factor with a chemical composition of semi‐volatile species (LO‐OOA and nitrate) and ammonium sulfate. The higher contribution of F4 is during the nighttime, which agrees with the findings from the previous section (Section 3.2) that the contribution of LO‐OOA increases during the night (MF > 0.30). The lower temperature during nighttime favors the condensation of these semi‐volatile species onto the newly formed particles, which could be the reason for the observed higher fraction of F4.

In addition to the size‐driven factors, two composition‐derived factors (F2 and F1) were also identified (Figure 5), which do not relate to any specific particle size ranges. F2 comprises nitrate (MF ∼ 0.08), sulfate (MF ∼ 0.00), ammonium (MF ∼ 0.01) and LO‐OOA (MF ∼ 0.90), indicating the dominance of semi‐volatile species in this factor. F1 comprises sulfate (MF ∼ 0.34), ammonium (MF ∼ 0.13) and MO‐OOA (MF ∼ 0.50), suggesting its highly oxidized and low‐volatile nature. These factors are assigned to composition‐driven because of the absence of a specific size range in the derived size factor profiles. F2 and F1 can be viewed as representations of bulk atmospheric composition, significantly contributed to by the formation and growth of new particles during the daytime. Overall, the results from the daytime hybrid analysis indicate that the initial stages of the nucleation involve ammonium sulfate, MO‐OOA and, then, later stages of growth are aided by HOA, LO‐OOA and nitrate.

**Figure 7:**
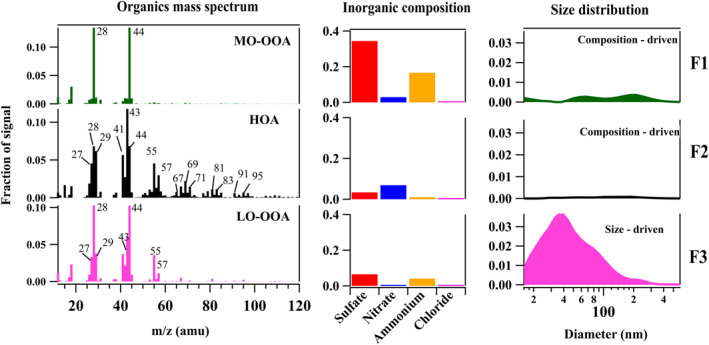
Hybrid positive matrix factorization‐derived factors (F1–F3) obtained for nighttime New particle formation events during Campaign 2. Each factor is split into three panels with separate axes. The panels on the left show the organic mass spectrum, the panels on the middle show the inorganic mass concentration (sulfate, nitrate, ammonium, chloride), and the panels on the right show the particle number size distribution. The factors are assigned as (F1) composition‐driven sulfate + ammonium + more‐oxidized OOA (MO‐OOA), (F2) composition‐driven nitrate + sulfate + ammonium + hydrocarbon like OA (HOA), (F3) size‐driven sulfate + ammonium + Less‐oxidized OOA (LO‐OOA).

The hybrid PMF analysis for the nighttime events during Campaign 2 is shown in Figure 7, where a three‐factor solution comprised of one size‐driven factor (F3) and two composition‐driven factors (F2 and F1) was obtained. The size‐driven factor F3 (Figure 7 and Table S3 in Supporting Information S1) represents the Aitken mode particles (mode ∼ 33 nm) with LO‐OOA (MF ∼ 0.76) and ammonium sulfate (MF ∼ 0.14 sulfate and MF∼ 0.09 ammonium) as the major components. The presence of sulfate with ammonium implies the presence of ammonium sulfate in F3. The composition driven factor F2 consists of HOA (MF ∼ 0.83), Sulfate (MF ∼ 0.05), Nitrate (MF ∼ 0.10) and ammonium (MF ∼ 0.01). Like daytime events, a composition‐driven factor (F1) of highly oxidized species was obtained. F1 comprise MO‐OOA (MF ∼ 0.32), sulfate (MF ∼ 0.43), nitrate (MF ∼ 0.04) and ammonium (MF ∼ 0.20).

**Figure 8:**
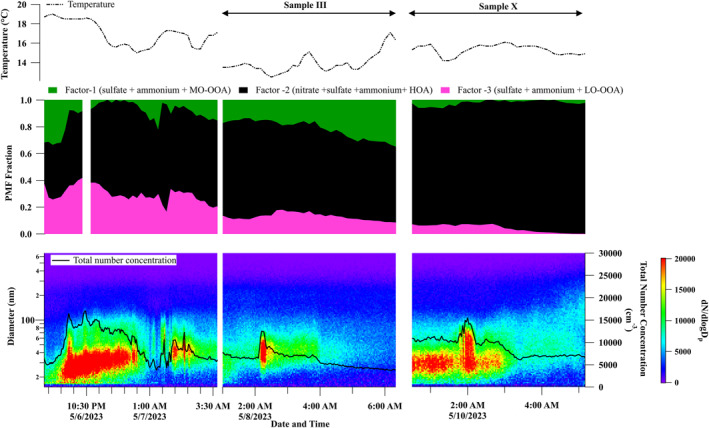
Time series of temperature (in degrees Celsius), fraction of hybrid PMF‐derived factors, particle number size distributions and total number concentrations for the nighttime events during Campaign 2.

Figure 8 depicts the temporal variation of aerosol number size distributions, the fraction of hybrid PMF‐derived factors, and temperature during nighttime events. An enhancement in the size‐driven factor‐3 is seen with the initiation of the event. This suggests the formation of ammonium sulfate and the condensation of semi‐volatile organic compounds, contributing to the Aitken mode in the PNSD. LO‐OOA is associated with initiating the event rather than MO‐OOA. This implies that low‐oxidized organic compounds, such as HOA and LO‐OOA, are abundant and condensed on the freshly formed Aitken mode particles. As time progressed, the contribution of F3 decreased, and F2 dominated and reached > MF ∼ 0.50 of the total fraction. F2 is a primary factor associated with local traffic/transport; the increase in this factor during the growth phase of the event can be viewed as the condensation of semi‐volatile organic compounds (LO‐OOA) on the primary particles. Supporting this conclusion, the temperature drops from the background to lower values during the start of the event, strongly favoring the condensation of semi‐volatile species. A gradual increase in the composition‐driven factor (F1) fraction with highly oxidized components was seen for the two events when the growth phase was completed, suggesting the probable conversion of the low‐oxidized compounds to more‐oxidized OOA. Similar to the observations from this study, contributions of LO‐OOA to NPF were reported by Nursanto et al. (2023) from a rural site in the Netherlands. In their study, the higher abundance of less‐oxidized organic compounds over more‐oxidized was attributed to the plausible emissions from the port of Rotterdam.

#### 3.4.2 Chemical Imaging of the Nighttime Events

**Figure 9:**
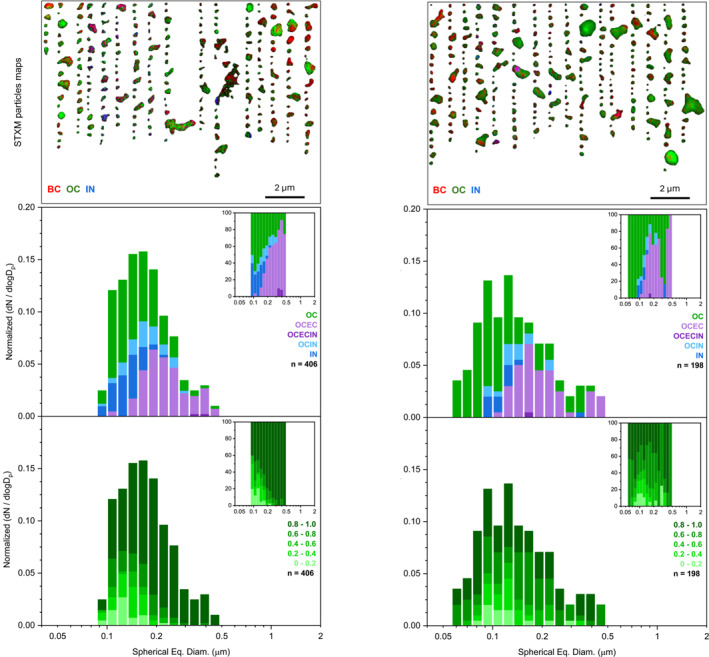
Upper panels: Representative carbon speciation maps derived from STXM/near‐edge X‐ray absorption fine structure analysis of individual particles from Samples III and X. Colors correspond to experimentally defined chemical components green–green‐organic carbon (OC), red–soot/elemental carbon (EC), and teal–inorganics (IN). Note that pixels can contain up to three components resulting in overlapping colors. Middle panels display size distributions of analyzed particles, which are shown as 8 bin per decade histograms in logscale to compare contributions from particles with different chemical components. Lower panels display same size distributions color‐coded to compare contributions from particles with varying organic volume fractions.

The chemical imaging STXM carbon speciation maps of individual particles collected on the nights of 5/8/2023 (Sample III) and 5/10/2023 (Sample X) further illustrate the internal mixing characteristics of particles during these nighttime NPF events. The upper panels of Figure 9 depict the assortment of particle morphologies and the complexity of particle internal heterogeneity. X‐ray absorption data acquired for the composition maps at the carbon K‐edge is separated into three components based on the NEXAFS spectral characteristics and applied threshold definition, whereby each pixel can contain components of OC (green), elemental carbon or soot (EC, red), and inorganic salts (IN, teal). If a particle composition map contains more than one or multiple components, then it will be assigned to one of the following categories: (a) OC, (b) IN, (c) OCEC, (d) OCIN, and (e) OCECIN. The middle panels of Figure 9 (and Figure S9 in Supporting Information S1) show the size‐resolved fractions of particles with different chemical mixing characteristics derived from the carbon component maps. The lower panels of Figure 9 show the size‐resolved organic volume fractions (OVF) of the same ensembles of particles, also calculated from their maps. Overall, the component mixing and OVF characteristics of particle ensembles corroborate AMS observations of Figure 8, indicating internal heterogeneity of individual particles, consistent with condensation of the secondary OC onto pre‐existing fine mode IN and EC/soot particles. The complex variation in particle morphologies and internal composition observed in particles likely arises from the dynamic interplay of primary emissions and secondary organic condensation during NPF events, reflecting a range of environmental conditions and chemical processes that influence particle formation and evolution over time (Chen et al., 2023; Pang et al., 2023; Tomlin et al., 2022). Of note, the significant contributions of EC/soot inclusions suggest their combustion‐related source, which would also be a source of OC emissions consistent with the HOA and LO‐OOA composition detected by AMS.

**Figure 10:**
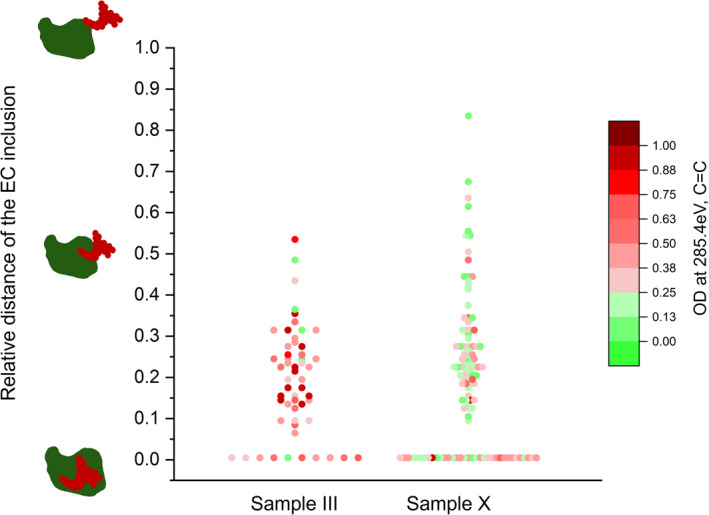
Radial distribution of EC (soot) inclusions with respect to organic carbon among analyzed particles derived from STXM/near‐edge X‐ray absorption fine structure measurements. Markers represent the measurement of individual particles. Violin plots display the density of individual measurements in Samples III, and X. The illustrations on the left represent the relative location of soot with respect to the entire particle.

The core‐shell relationship between EC/soot and condensing OC components can be further confirmed from additional processing of particle chemical images, featuring identification of the EC/soot position from the particle center, calculated relative to the longest distance between the center to the edge of the entire host particle (see Text S4 in Supporting Information S1 for details). Figure 10 illustrates the distribution of EC/soot inclusions within their respective host particles for OCEC and OCECIN mixing classes, derived from the particle chemical images from samples III and X. Colors of individual symbols additionally indicate values of the optical density at 285.4 eV (OD_285.4 eV_), which are associated with the C = C sp^2^ absorbance indicative of mass fractions of EC within particles. In both NPF episodes, particle images indicate substantial clustering of EC/soot inclusions in the center or close to it, confirming their mixing characteristics consistent with the OC condensation onto pre‐existing EC particles.

The results of the hybrid PMF analysis and the particle images indicate the involvement of multiple species in NPF events. This is consistent with other studies where the contribution of organic species to the growth of nucleated particles has been reported using size‐resolved AMS studies (Setyan et al., 2014). Q. Zhang et al. (2004) reported that the growth of the nucleating particles was sustained by the condensation of oxygenated organic species at the Pittsburg supersite, while Setyan et al. and Vakkari et al. reported similar findings in sub‐urban location in California (Setyan et al., 2014) and a rural site in South Africa (Vakkari et al., 2015), respectively.

More studies are required from this region involving the measurements of Nucleation mode particles and VOC concentrations to understand the contributing sources and the formation pathways of NPF. Future longer time series and climatology of NPF data are essential to improve the understanding of NPF and its impact on climate and human health.

## 4 Summary and Conclusions

This study investigated the processes and species involved in NPF events over a semi‐urban location in Rehovot, Israel, using concurrent observations of PNSDs, non‐refractory submicron aerosol chemical composition, and meteorological conditions made during the summer of 2023 and 2021 combined with a hybrid–source apportionment approach. The major findings of the study are the following:

1. Several NPF events were observed, resulting in a large reservoir of nucleation and Aitken mode particles. The events are well‐defined nucleation events during the daytime. In contrast, the nighttime events were mostly disturbed by large background concentrations.
2. The source apportionment of OA revealed a 4‐factor solution during 2021 and a 5‐factor solution during 2023. HOA and BBOA constitute the primary factors, whereas secondary OA consists of LO‐OOA and MO‐OOA. The BBOA factor was only seen during the national bonfire festival (Lag Ba'Omer event), indicating the absence of any biomass‐burning sources in the background.
3. An enhancement in the concentration of sulfuric acid proxy (average concentration ∼ 2 × 10^6^ to 12 × 10^6^ molecules/cm^3^) was estimated based on observations in the daytime NPF events, suggesting the photochemical formation of sulfuric acid and its role in nucleation and further growth.
4. A large influx of the Aitken mode particles was observed during the onset of the biomass burning event and it subsequently evolved toward the accumulation mode with atmospheric aging, as evidenced by increasing O/C ratios (O:C changed from 0.4 to 0.6 during 2023 and 0.3 to 0.7 during 2021).
5. The hybrid PMF analysis revealed both size‐driven (three factors during Daytime and one factor during nighttime representing nucleation and growth) and composition‐driven factors (two factors during daytime and nighttime).
6. A simultaneous enhancement in size‐driven factor (F5 factor having a possible chemical makeup of sulfate, ammonium and MO‐OOA) and sulfuric acid proxy was seen along with daytime NPF events, strongly suggesting the involvement of multiple components, including more oxidized organics in the nucleation. HOA, LO‐OOA and nitrate aided the growth of newly formed particles.
7. Nighttime events were associated with an enhancement in the size‐driven factor—F3 with a higher background of composition‐driven factor ‐ F2 (mainly comprising ammonium sulfate, LO‐OOA, HOA, and nitrate), suggesting the involvement of semi‐volatile organic compounds in the formation and growth of particles during night.
8. The results from the chemical imaging indicated internal heterogeneity of individual particles, consistent with condensation of the secondary OC onto pre‐existing fine mode inorganics including elementary carbon/soot particles.

## Supporting information

Supporting Information S1

## Data Availability

The data used for making the figures can be downloaded at the institute repository at (Rudich, 2024). All the figures in the manuscript were made using Igor Pro (https://www.wavemetrics.com/). NCEP/NCAR reanalysis data for Figure S1 in Supporting Information S1 is available on the website: https://www.ready.noaa.gov/data/archives/reanalysis/. The software used for AMS data analysis can be downloaded at: https://cires1.colorado.edu/jimenez‐group/ToFAMSResources/ToFSoftware/. The code utilized in the PMF analysis is part of the PMF Evaluation Tool (PET) v2.03 written as Igor procedures, available at https://cires1.colorado.edu/jimenez‐group/wiki/index.php/PMF‐AMS_Analysis_Guide#PMF_Evaluation_Tool_Software.
