# Supplementary material for: Investigating New Particle Formation and Growth Over an Urban Location in the Eastern Mediterranean
Source: J Geophys Res Atmos. 2024 Nov 30;129(23):e2024JD041802. doi: 10.1029/2024JD041802 (PMC11608121; doi:10.1029/2024JD041802)
Supplement: Supplementary file 1 — Supporting Information S1 [file JGRD-129-0-s001.docx]

*Journal of Geophysical Research, Atmospheres*

Supporting Information for

**Understanding the New Particle Formation Pathways over an Urban Location in the Eastern Mediterranean**

Ajith T C^1^, Eli Windwer^1*^, Chunlin Li^2^, Zheng Fang^1^, Sobhan Kumar Kompalli^3^, Farhan R Nursanto^4^, Temitope E. Olayemi^5^, Julius I. Ese^5^, Steven A.L. Sharpe^5^, Matthew Fraund^6^, Ryan C. Moffet*^7^*, Alexander Laskin*^5,8^*, Juliane L Fry^4^, and Yinon Rudich^1*^

^1Department of Earth and Planetary Sciences, Weizmann Institute of Science, Rehovot, Israel^

^2College of Environmental Science and Engineering, Tongji University, Shanghai, China^

^3Space Physics Laboratory, Vikram Sarabhai Space Centre, Thiruvananthapuram, India^

^4Meteorology and Air Quality (MAQ), Environmental Sciences Group,^

^Wageningen University and Research (WUR), Wageningen, Netherlands^

^5Department of Chemistry, Purdue University, West Lafayette, IN 47907, USA^

^6Fraund Consulting, Pleasant Hill, California, CA 94523, USA^

^7Sonoma Technology, Inc., Petaluma, CA 94954, USA^

^8Department of Earth Atmospheric and Planetary Sciences, Purdue University, West Lafayette, IN 47907, USA.^

^*Corresponding author^

**Corresponding authors**: Yinon Rudich ([yinon.rudich@weizmann.ac.il](mailto:yinon.rudich@weizmann.ac.il)), Eli Windwer (eli.windwer@weizmann.ac.il)

**Contents of this file**

1. Isentropic five-day air mass back trajectories arriving 500 m above the observational site at Rehovot, Israel, during Campaign 1 and Campaign 2 (Figure S1).
2. Note on the STXM measurements (Supplemental texts S1 to S4)
3. Mass spectral profile of PMF-derived factors (Figures S2 to S3)
4. Mean meteorological and aerosol parameters measured during the observation period (Table S1 to S2)
5. Mass fraction of aerosol species contributing towards the derived hybrid PMF factors (Table S3)
6. Detailed note on the hybrid PMF analysis (Supplemental text S5)
7. Down weighting constant for the hybrid PMF analysis (Table S4)
8. Diagnostic plots for hybrid PMF analysis (Figures S4-S8)
9. STXM-derived values of particles, showing the number of particle size distributions (PSD) of Sample III and Sample X (Figure S9).

**Introduction**

This document includes the figures, tables, and notes to support the methodology and the results. Figures include isentropic five-day air mass back trajectories arriving 500 m above the observational site at Rehovot, Israel, during (a) Campaign 1 and (b) Campaign 2 (Fig. S1). The 4-factor PMF analysis solution obtained for campaign-1 is shown in Figure S2, and the 5-factor solution for campaign-2 is shown in Figure S3. A detailed note on the STXM measurements, calculation of Organic Volume Fraction (OVF) of analyzed particles from STXM-NEXAFS data, Spherical equivalent diameter (SED) calculations and ‘EC to the center’ calculations are given in supplemental texts S1- S4. The mean meteorological parameters for the two campaigns are listed in Table S1. Table S2 provides the mean aerosol and meteorological parameters during the daytime and nighttime. In Table S2, the data points for campaign 1 and campaign 2 are combined, and the average is taken for the entire dataset. A detailed note on the hybrid PMF analysis is given in the supplemental text S1. The downweighting constant used for hybrid analysis and the calculation is given in Table S4. The diagnostic plots, including the time series of total reconstructed mass, residual, and scaled residual for the hybrid PMF analysis performed for the daytime and nighttime, are shown in Figures S4 and S5. Figure S6 shows the scatter plot between Q/Qexpected and number of factors for daytime and nighttime events. Figure S7 shows the scatter plot between Q/Qexpected and fpeak parameter for daytime and nighttime events. Figure S8 shows the scatter plot between Pearson uncentered R timeseries vs Pearson R profiles for daytime and nighttime. Figure S9 shows the STXM-derived values of particles, showing the number of particle size distributions (PSD) of Sample III (Top) and Sample X(Bottom).


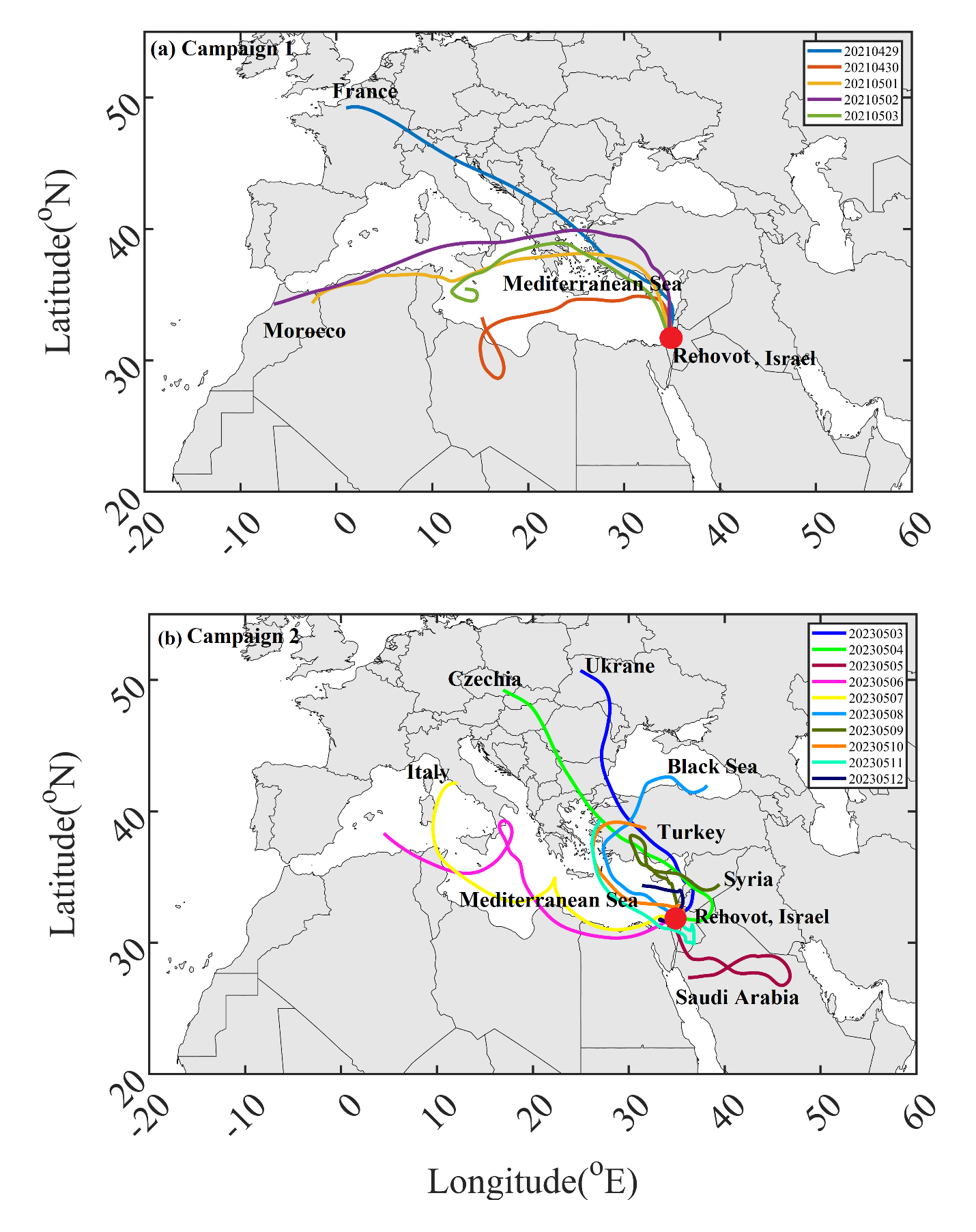


Figure S1: Isentropic five-day air mass back trajectories arriving 500 m above the observational site at Rehovot, Israel, during (a) Campaign 1 and (b) Campaign 2.

Supplemental Text S1. STXM measurements and particle-type mapping

Scanning Transmission X-ray Microscopy coupled with Near-Edge X-ray Absorption Fine Structure (STXM/NEXAFS) is a powerful technique for analyzing the chemical environment of elements within a sample. It utilizes high-resolution X-rays focused on specific element absorption edges, like the carbon K-edge. By analyzing the absorption features at this edge, we can differentiate between different types of carbon bonding(Laskin et al., 2019; Signorell & Reid, 2010).

Our measurements were conducted at beamline 5.3.2.2 of the Advanced Light Source at Lawrence Berkeley National Laboratory. The STXM chamber was backfilled with helium (He) and maintained at a pressure of ~500 Torr before data acquisition. To capture information across the carbon K-edge, images were acquired at various photon energies while raster scanning the sample through the focused X-ray beam.(Tomlin et al., 2022) These images, referred to as "carbon maps" (C_maps_), provide a fast and statistically robust way to analyze large numbers of particles. C_maps_ offers a detailed view with a field-of-view of approximately 15×15 μm, a pixel resolution of 35 nm, and a dwell time of 1 millisecond per pixel. Images are acquired at four key energies: 278 eV (pre-edge), 285.4 eV (C=C bonds, associated with elemental carbon - EC), 288.5 eV (-COOH bonds, associated with organic carbon - OC), and 320 eV (post-edge).(Tomlin et al., 2022)

Using previously defined thresholds, individual pixels within the C_maps_ are assigned as organic carbon (OC), elemental carbon (EC), or inorganic material (IN) based on the dominant carbon bonding environment detected.(Tomlin et al., 2022) This allows for the creation of maps where each pixel represents the dominant carbon type within that specific location of the particle. Furthermore, mixed-component pixels are categorized as OC, OCEC (organic and elemental carbon), OCECIN (organic, elemental, and inorganic carbon), OCIN (organic and inorganic carbon), or IN. This comprehensive analysis enables the estimation of the relative abundance and distribution of OC, EC and IN within particles.

**Supplemental Text S2: Organic Volume Fraction (OVF) of analyzed particles from STXM-NEXAFS data.**

OVF is a metric representing the relative volume of organic material within a particle. For the calculation of the OVF, the thicknesses of both the organic and inorganic components within each pixel can be calculated from the average optical densities at the pre- and post-edge ratios of each particle.(Fraund et al., 2019; O’Brien et al., 2015)

To quantify the X-ray absorption within each pixel, the intensity image is converted into an optical density (OD) image on a pixel-by-pixel basis using the following equation:

$$OD= -\ln\left( \frac{I}{I_{o}} \right)= \mu\rho t$$

OD is optical density, *I* is the intensity of the given pixel, and *I*_o_ is the background intensity, with *μ* being the mass absorption coefficient, *ρ* being the density, and *t* being the thickness of the given pixel.(Fraund et al., 2019)

To estimate the thickness of carbon and inorganic components, the optical densities (OD) at 320 and 278 eV were used.(O’Brien et al., 2015)

$$\mathrm{OD}_{278}=\mu_{278}^{\mathrm{in}}\rho^{\mathrm{in}}t^{\mathrm{in}}+\mu_{278}^{\mathrm{org}}\rho^{\mathrm{org}}t^{\mathrm{org}}$$

$$\mathrm{OD}_{320}=\mu_{320}^{in}\rho^{\mathrm{in}}t^{\mathrm{in}}+\mu_{320}^{\mathrm{org}}\rho^{\mathrm{org}}t^{\mathrm{org}}$$

In and Org represent inorganic and organic components, and $X^{\mathrm{in}}=\mu_{320}^{\mathrm{in}}/\mu_{278}^{\mathrm{in}}$

For atmospheric particles, the OD at a given energy is estimated as a linear combination of the ODs of the inorganic and organic components.(O’Brien et al., 2015) By taking OD_320_-OD_278_, the thickness of the inorganic (*t*_in_) and organic (*t*_org_) components can be calculated as:

$t^{\mathrm{Org}}= \frac{\mathrm{OD}_{320}-X^{\mathrm{in}}{OD}_{278}}{\left( \mu_{320}^{\mathrm{org}}-X^{\mathrm{in}}\mu_{278}^{\mathrm{org}} \right)\rho^{\mathrm{org}}}$

$t^{I}= \frac{\mathrm{OD}_{278}-\mu_{278}^{\mathrm{org}}\rho^{\mathrm{org}}t^{\mathrm{org}}}{\mu_{278}^{\mathrm{in}}\rho^{\mathrm{in}}}$

The OVF for each pixel is calculated as the ratio of the organic thickness to the total thickness of the particle.(O’Brien et al., 2015)

$$\mathrm{OVF}= \frac{t^{\mathrm{org}}}{t^{\mathrm{org}}+ t^{\mathrm{in}}}$$

**Supplemental Text S3: Spherical equivalent diameter (SED) calculations**


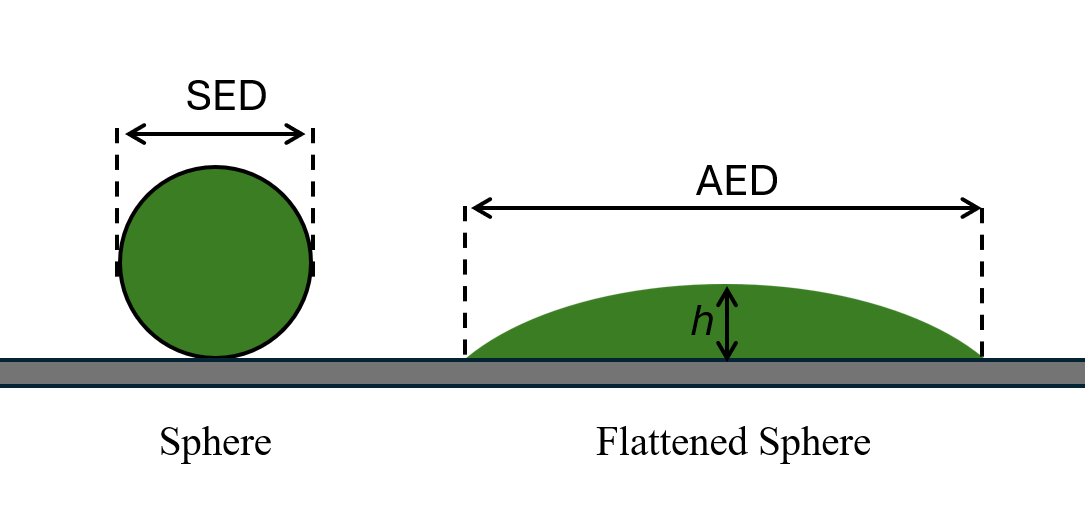


When particles collide with a substrate, their shapes are often transformed from their original form, and the impact creates flattened morphologies that closely resemble dome-like hemispheres. These domes can be characterized by two key dimensions: their base diameter (*D*) and their height (*h*).(Laskin et al., 2006) To estimate the volume of these individual particles, we can leverage a well-established mensuration formula which will provide a more accurate estimate of the particle's volume based on its easily measurable base diameter and height. This approach allows us to quantify the three-dimensional space occupied by each particle after impacting the substrate.(Laskin et al., 2006) For an accurate estimation of the height-to-diameter ratio, the height of each particle can be calculated by relating the total carbon absorption (TCA) to the height.(Rivera-Adorno et al., 2023)

$$Height, h= \frac{TCA}{0.68}$$

$$V_{particle}=\frac{\pi h}{6}\left( h^{2}+ \frac{3D^{2}}{4} \right)$$

 Where *D* is the 2D projection area equivalent diameter (AED) determined from particle images; *h* is the height estimated from TCA measurement. The SED can then be deduced by relating the volume of the particle to the size of a sphere.

$$SED=\left( \frac{6 \times V_{particle}}{h} \right)^{\frac{1}{3}}$$

**Supplemental Text S4.** ‘EC to the center’ calculations


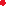


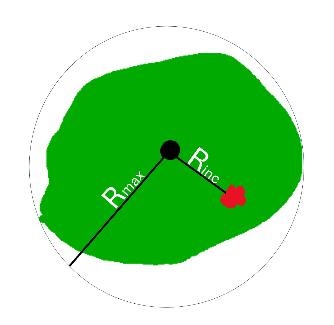


To compare where an EC/soot inclusion sits inside different-sized particles, the distance from the EC inclusion to the particle's center, R_inc_ is divided by the longest possible distance within the particle, R_max_ (center to edge). This gives a ratio between 0 and 1. A ratio of 1 means the soot inclusion is as far away from the center as possible, no matter how big or small the particle is. With this method, we can easily compare the relative location of the soot inclusion across different-sized particles.(Moffet et al., 2016)


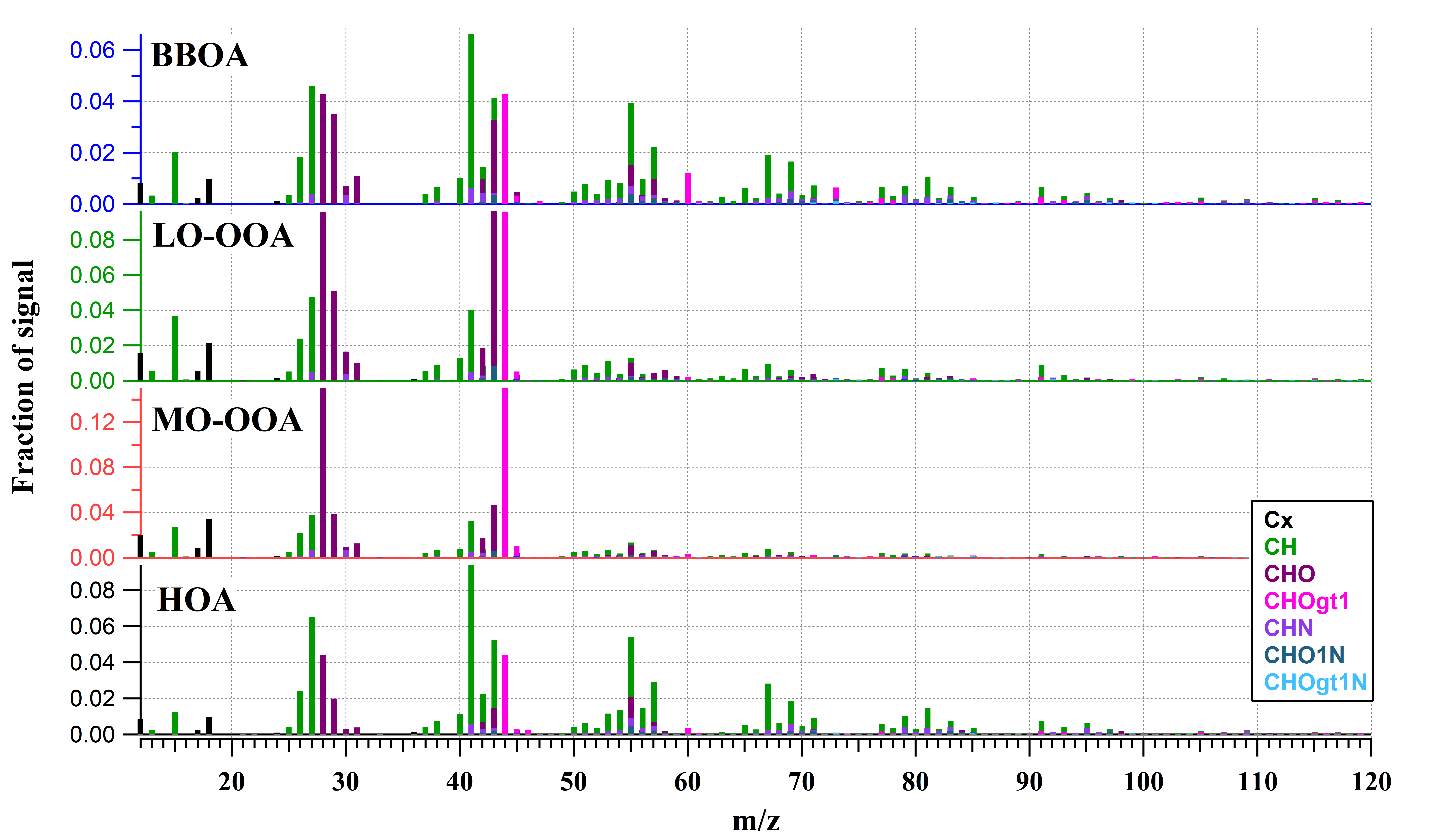


**Figure S2:** The mass spectral profile of different factors of organic aerosols derived from the PMF analysis of the AMS data during campaign 1


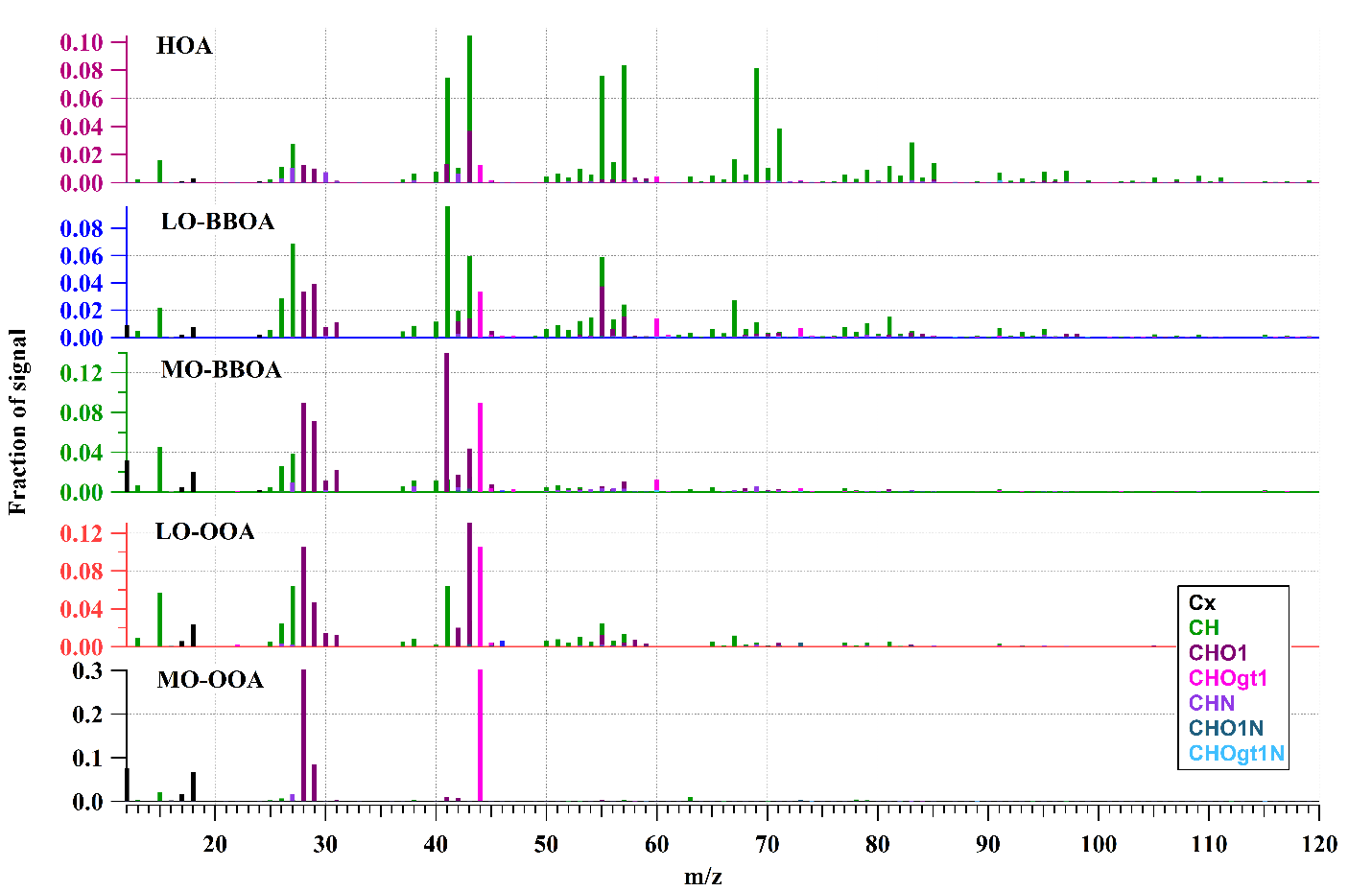


**Figure S3:** The mass spectral profile of different factors of organic aerosols derived from the PMF analysis of the AMS data during campaign 2.

Table S1: Mean meteorological parameters measured during the campaigns.

|  | **Campaign 1** | | | **Campaign 2** | | |
| --- | --- | --- | --- | --- | --- | --- |
|  | **Average ± SD** | **Maximum** | **Minimum** | **Average ± SD** | **Maximum** | **Minimum** |
| **Temperature (°C)** | 22.9 ± 4.9 | 30.6 | 13.6 | 21.1 ± 4.7 | 38.6 | 12.1 |
| **Relative Humidity (%)** | 55.5 ± 19.1 | 93 | 21 | 64.6 ± 17.5 | 94.1 | 10 |
| **Wind Speed (m/s)** | 2.20 ± 1.2 | 5.3 | 0.1 | 2.0 ± 1.3 | 6.9 | 0.1 |

Table S2: Mean values for meteorological, and aerosol parameters separated for daytime and nighttime events.

|  | **Day time Events** | **Nighttime Events** |
| --- | --- | --- |
| Temperature (°C) | 24.7 ± 4.9 | 16.0 ± 2.2 |
| Relative Humidity (%) | 47.7 ± 18.6 | 73.2 ± 12.4 |
| Wind Speed (m s^-1^) | 2.6 ± 1.3 | 1.0 ± 0.6 |
| Geometrical Mean Diameter (nm) | 57.3 ± 10.8 | 53.5 ± 9.5 |
| Total number concentration (cm^-3^) | 8946 ± 4607 | 7676 ± 2427 |
| Mass fraction of Sulfate | 23.6 % | 12.0 % |
| Mass fraction of Nitrate | 5.1 % | 5.4 % |
| Mass Fraction of HOA | 13.1 % | 18.7 % |
| Mass Fraction of MO-OOA | 28.1 % | 14.7 % |
| Mass Fraction of LO-OOA | 19.6 % | 41.7 % |

Table S3: Mass fraction of each aerosol species contributing towards the derived hybrid PMF factors for the daytime and nighttime events during campaign 2.

|  | **Daytime events** | | | | | **Nighttime events** | | |
| --- | --- | --- | --- | --- | --- | --- | --- | --- |
|  | **F5** | **F4** | **F3** | **F2** | **F1** | **F3** | **F2** | **F1** |
| **Organics (mass fraction)** | 0.42 | 0.44 | 0.78 | 0.90 | 0.51 | 0.76 | 0.83 | 0.32 |
| **Sulfate (mass fraction)** | 0.40 | 0.27 | 0.14 | 0.00 | 0.34 | 0.14 | 0.05 | 0.43 |
| **Nitrate (mass fraction)** | 0.00 | 0.12 | 0.00 | 0.09 | 0.01 | 0.00 | 0.10 | 0.04 |
| **Ammonium (mass fraction)** | 0.18 | 0.16 | 0.07 | 0.01 | 0.14 | 0.09 | 0.01 | 0.20 |
| **Chloride (mass fraction)** | 0.00 | 0.01 | 0.00 | 0.00 | 0.00 | 0.00 | 0.01 | 0.01 |

Supplemental Text S5. Hybrid PMF Analysis

Hybrid PMF analysis combines the PNSD measurements with concurrent aerosol chemical composition data obtained from AMS (Nursanto et al., 2023). In the hybrid PMF approach, the 10-min average of organic mass spectra in the mass-to-charge ratio (m/z) range of 12 to 120 were combined with inorganic mass concentrations (sulfate, ammonium, nitrate, and chloride), and 10-min averaged particle number concentrations (dN) across 28 diameter size bins, derived from a total of 105 size bins of the SMPS. Each bin consist of the sum of 4 concentration points (raw size bins used and median diameter of the summed sizes (for campaign 2): 15.1-16.8 (median=16nm), 17.5-19.5 (median=18.45nm), 20.2-22.5 (median = 21.3nm), 23.3-25.9 (median=24.55nm), 26.9-30 (median=28.4nm), 31.1-34.6 (median=32.8nm), 35.9-40 (median=37.85nm), 41.4-46.1 (median=43.7nm), 47.8-53.3 (median=50.5nm), 55.2-61.5 (median=58.35nm), 63.8-71 (median=67.3nm), 73.7-82 (median=77.75nm), 85.1-94.7 (median=89.8nm), 98.2-109.4 (median=103.65nm), 113.4-126.3 (median=119.75nm), 131-145.9 (median=138.25nm), 151.2-168.5 (median=159.65nm), 174.7-194.6 (median=184.4nm), 201.7-224.7 (median=212.9nm), 232.9-259.5 (median=245.85nm), 269-299.6 (median=283.9nm), 310.6-346 (median=327.9nm), 358.7-399.5 (median=378.6nm), 414.2-461.4 (median=437.25nm), 478.3-532.8 (median=504.9nm), 552.3-615.3 (median=583nm), and 637.8-661.2 (median=649.5nm). The error matrix for the organic mass spectrum and the minimum error (minErr) of all species was generated by the standard AMS data analysis software (Squirrel). The error for inorganics and number size distributions are taken to be the standard deviation of the raw data.

The variables used for creating the input matrix are in different magnitudes and units (organic mass spectra, inorganic mass concentration (different magnitude but same units compared to organic mass spectrum) and number concentrations (different magnitude and units compared to organic mass spectrum) in 28 size bins). Therefore, the inorganic mass concentrations and number size distributions (28 size bins) are downweighted to have similar magnitudes over others. For this purpose, the values (inorganics and number size distribution) in the input data matrix and error matrix were downweighted by dividing with a downweighting constant (DWC) (given in Table S4). The idea behind using the DWC is described in Nursanto et al., 2023. Further, the default downweighting procedures provided by PETv3.04 are also applied for the duplicated ions (m/z 44, 28, 18, 17 and 16 signals in the organic mass spectrum).

Table S4. The variables used for down weighting constant (DWC).

| Variable | Value (Campaign 2) |
| --- | --- |
| DWC inorganics = $\frac{C_{SO4, 95\%}}{C_{f44, 95\%}}$ | 1.57 |
| DWC dN $=20 \times\frac{C_{dN, 95\%}}{C_{f44, 95\%}}$ | 57498 |

The analysis for each campaign was performed by running the PMF model in the unconstrained mode for 5 p values (number of factors). The optimum p-value was selected based on the lowest residual, minimum Q/Q expected and the correlation between factors (time series and spectral profiles). Further, the rotational ambiguity of the solution was explored by varying fpeak from -1 to +1 with a delta value of 0.1. A 5-factor solution was selected for the daytime with an fpeak value of 0.20 and a 3-factor solution for nighttime with an fpeak value of 0.10. The diagnostic plots for the hybrid PMF analysis are given in, Figures S4-S8.


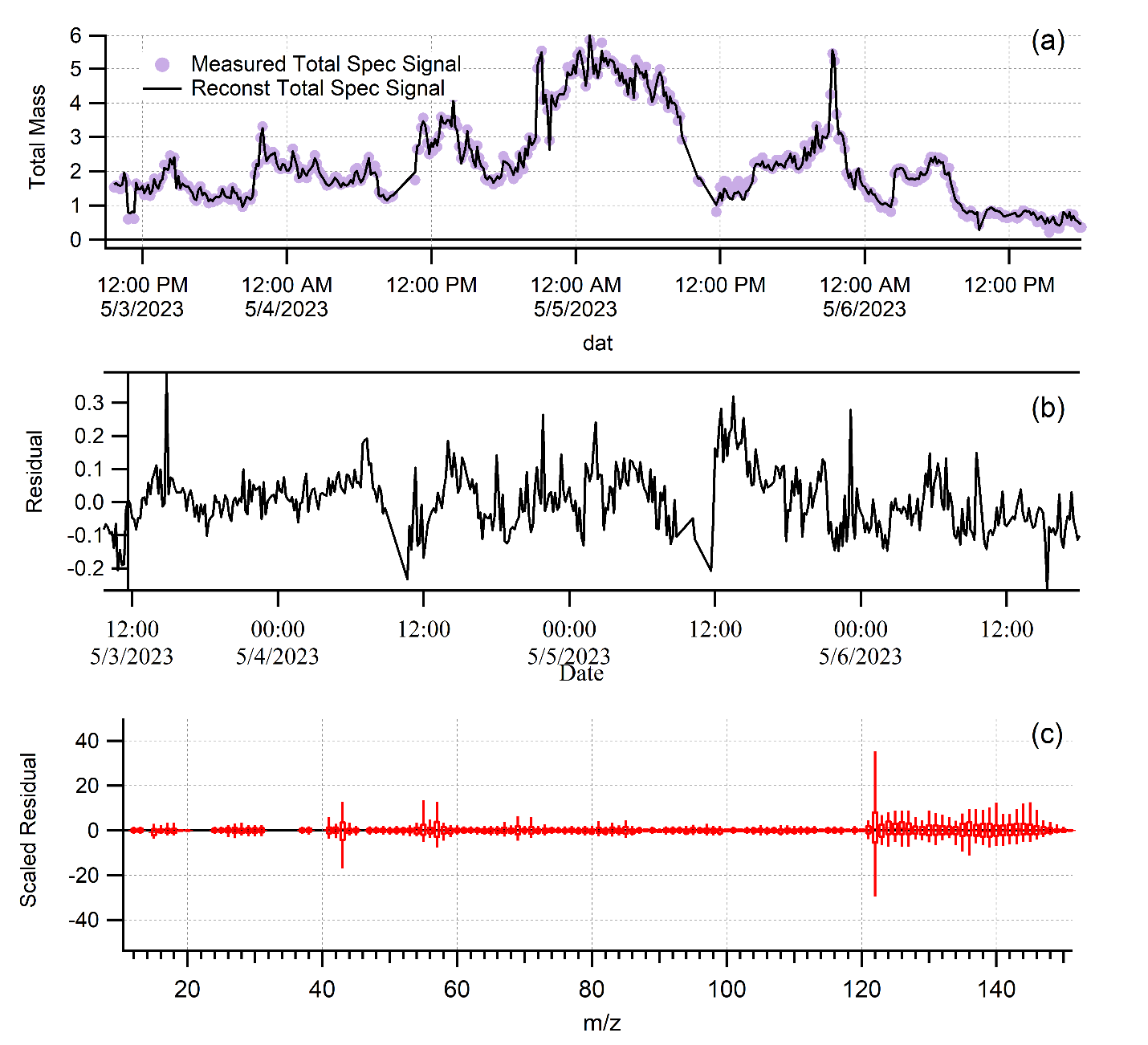


Figure S4: Diagnostic plot of daytime hybrid PMF analysis. (a) time series of measured and reconstructed PMF mass, (b) time series of the residual of the least square fit, and (c) distribution of scaled residuals for each m/z


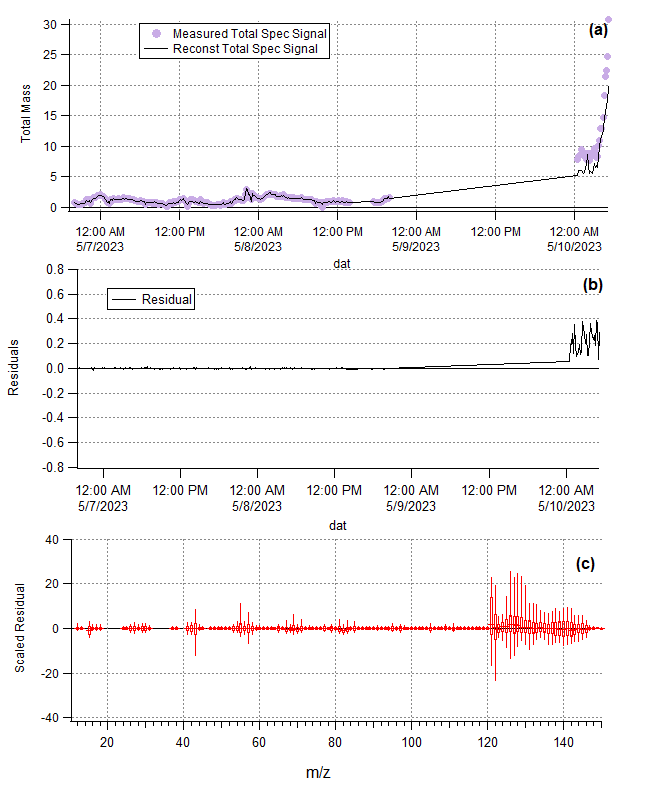


Figure S5: Diagnostic plot of nighttime hybrid PMF analysis. (a) time series of measured and reconstructed PMF mass, (b) time series of the residual of the least square fit, and (c) distribution of scaled residuals for each m/z


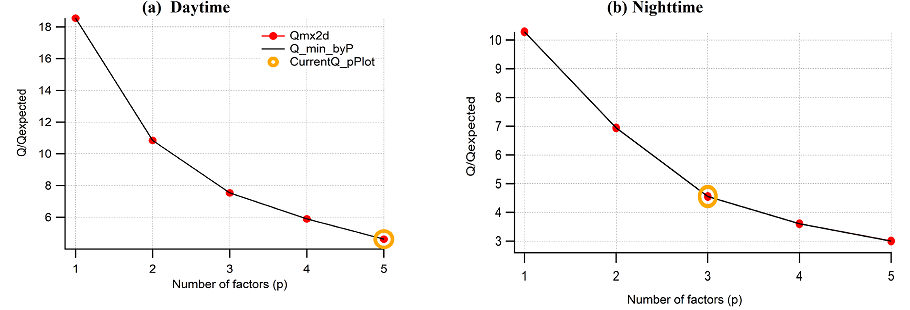


Figure S6: Diagnostic plot. Q/Qexpected vs number of factors for (a) daytime and (b) nighttime.


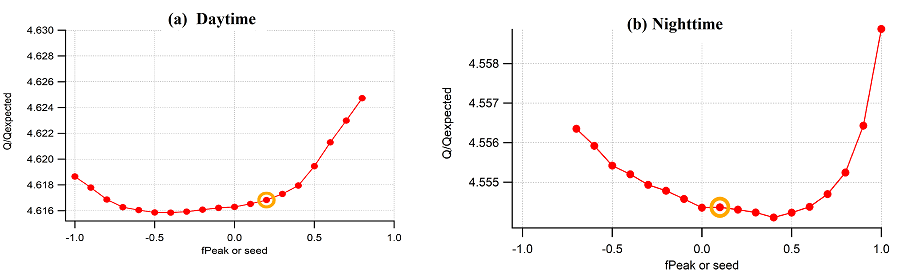


Figure S7: Diagnostic plot. Q/Qexpected vs fpeak for (a) daytime and (b) nighttime.


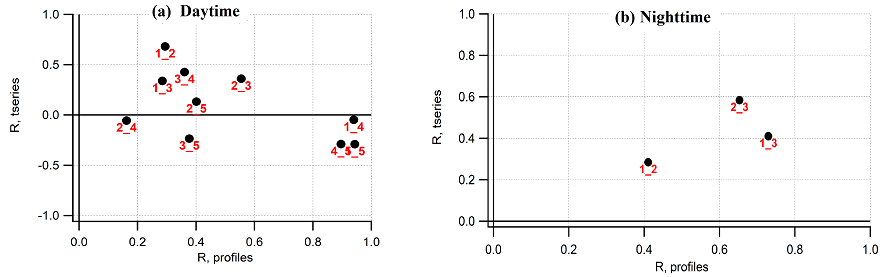


Figure S8: Diagnostic plot. Pearson uncentered R timeseries vs pearson R profiles for (a) daytime and (b) nighttime.


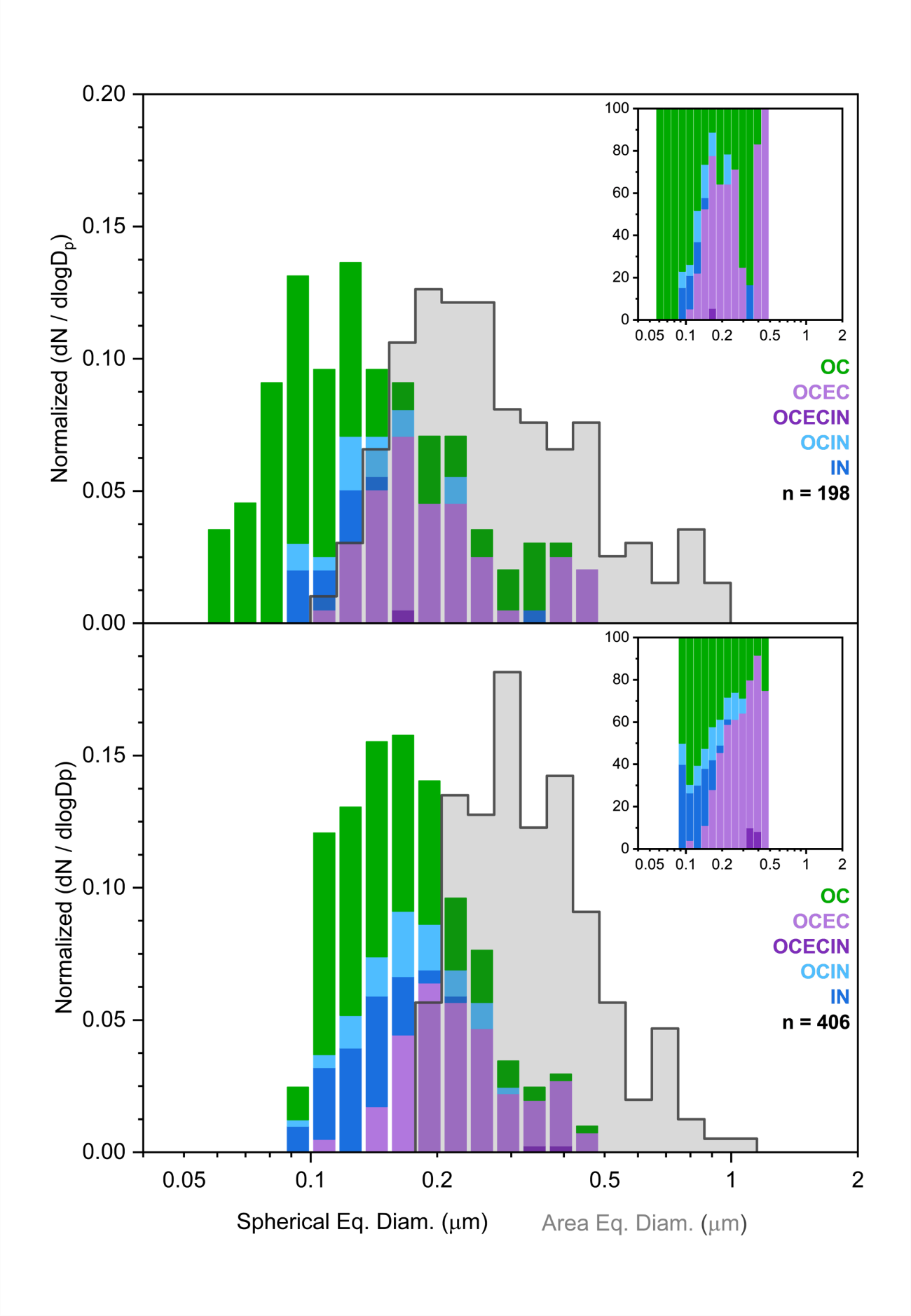


Figure S9: STXM-derived values of particles showing the number particle size distribution (PSD) of Sample III (Top) and Sample X(Bottom). The colored histogram shows spherical equivalent diameters (SED) calculated from STXM images of particles, while grey histogram *represents the same dataset of area equivalent diameters (AED)*
